# Evaluation of the uncertainty in calculating nanodosimetric quantities due to the use of different interaction cross sections in Monte Carlo track structure codes

**DOI:** 10.1371/journal.pone.0340500

**Published:** 2026-01-09

**Authors:** Carmen Villagrasa, Giorgio Baiocco, Zine-El-Abidine Chaoui, Michael Dingfelder, Sébastien Incerti, Pavel Kundrát, Ioanna Kyriakou, Yusuke Matsuya, Takeshi Kai, Alessio Parisi, Yann Perrot, Marcin Pietrzak, Jan Schuemann, Hans Rabus

**Affiliations:** 1 Autorité de sÛreté nucléaire et de radioprotection (ASNR), PSE-SANTE/SDOS/LDRI, F-92260, Fontenay aux Roses, France; 2 Radiation Biophysics and Radiobiology Group, A. Volta Physics Department, University of Pavia, Pavia, Italy; 3 Physics Department, Faculty of Sciences, Ferhat Abbas University Setif1. Algeria,; 4 Department of Physics, East Carolina University, Greenville, North Carolina, United States of America; 5 Université de Bordeaux, CNRS, LP2I, UMR, Gradignan, France; 6 Department of Radiation Dosimetry, Nuclear Physics Institute of the CAS, Na Truhlářce, Czech Republic; 7 Medical Physics Laboratory, Department of Medicine, University of Ioannina, Ioannina, Greece; 8 Faculty of Health Sciences, Hokkaido University, Sapporo, Hokkaido , Japan; 9 Nuclear Science and Engineering Center, Japan Atomic Energy Agency, 2-4 Shirane Shirakata, Tokai, Ibaraki , Japan; 10 Department of Radiation Oncology, Mayo Clinic, Jacksonville, Florida, United States of America; 11 Cyclotron Centre Bronowice, The Henryk Niewodniczanski Institute of Nuclear Physics Polish Academy of Sciences, Radzikowskiego, Krakow, Poland; 12 Radiological Metrology and Biomedical Physics Division, Nuclear Equipment and Technology Department, National Centre for Nuclear Research, Otwock-Swierk, Poland; 13 Department of Radiation Oncology, Massachusetts General Hospital & Harvard Medical School, Boston, Massachusetts, United States of America; 14 Physikalisch-Technische Bundesanstalt (PTB), Abbestrasse 2-12, Berlin, Germany; Cameroon National Radiation Protection Agency, CAMEROON

## Abstract

Biological effects induced by diverse types of ionizing radiation are known to show important variations. Nanodosimetry is suitable for studying the link between these variations and the patterns of radiation interactions within nanometer-scale volumes, using experimental techniques complemented by Monte Carlo track structure (MCTS) simulations. However, predicted nanodosimetric quantities differ among MCTS codes, primarily because each code employs distinct molecular-scale particle interaction models. This multi-code study examines these variations for low-energy electrons (20–10,000 eV), which play a critical role in energy deposition and biological effects by virtually all types of ionizing radiation. Specifically, the hypothesis tested in this work is that inter-code variability in nanodosimetry results is mainly caused by differences in assumptions regarding total interaction cross sections. Ionization cluster size distributions and derived nanodosimetric parameters were simulated with seven MCTS codes (PARTRAC, PHITS-TS, MCwater, PTra, and three Geant4-DNA options) in liquid water as a surrogate for biological tissue. Significant inter-code differences were observed, especially at the lowest energies. They were substantially reduced upon replacing the original cross sections in each code with a common, averaged dataset, created *ad-hoc* for this study and not based on theoretical assumptions. For example, for 50 eV electrons in 8 nm spheres, the variability in the predicted mean ionization numbers decreased from 23% to 5%, and in the probability of inducing two or more ionizations from 34% to 7% (relative standard deviations). This quantification demonstrates that total interaction cross sections are the primary source of uncertainty at low electron energies. A sensitivity test using DNA damage simulations with the PARTRAC code revealed that cross section variations notably affect biological outcome predictions. Replacing the code’s original cross sections with the averaged ones increased the predicted double-strand break yield by up to 15%. These findings underscore the urgent need for improved characterization of low-energy electron interaction cross sections to reduce uncertainties in MCTS simulations and enhance mechanistic understanding of radiation-induced biological effects.

## Introduction

Ionizing radiation has both positive and negative effects on biological systems. It is widely used in medicine for diagnostics (e.g., X-rays, CT, PET) and treatment (e.g., radiotherapy for cancer). It also plays a role in industry (e.g., defectoscopy) and scientific research. Radiation protection aims to minimize harmful effects as much as reasonably achievable.

Different types of ionizing radiation lead to different biological effects. Recognizing these differences is essential in fields such us ion beam radiotherapy [1,2], nuclear medicine [3], and space radiation protection [4–8]. Radiation quality can be described using macroscopic measures such as linear energy transfer (LET), or more detailed microscopic and nanoscopic approaches. The microscopic approach considers radiation effects at the length scale of a cell, its nucleus or organelles scale (about 0.1–10 µm) and uses microdosimetric quantities such as lineal energy distributions [9–12]. At nanometric scales, nanodosimetry [13,14] aims to describe radiation quality by physical radiation effects relevant to cellular deoxyribonucleic acid (DNA) damage, especially double-strand breaks (DSBs), and the induction of cell death [15].

Mechanistic models and simulations have helped explain how different types of radiation cause various biological effects. These models start at the molecular level, tracking how energy deposition causes DNA damage, and extend to predict broader biological outcomes like mutations or cell inactivation [1,16–24]. In general, mechanistic approaches rely on numerical simulations. The first step is to simulate how ionizing radiation interacts with biological materials at the nanometer level. These interactions transfer energy, causing molecular damage, particularly to nuclear DNA. The induced damage pattern depends on the structure of the DNA and chromatin and on the spatial distribution of energy transfer points known as the particle track.

Monte Carlo track structure (MCTS) simulations are used for this purpose. An overview of corresponding MTCS codes can be found in [25]. These differ from conventional condensed-history Monte Carlo simulations of radiation transport, which group together a large number of individual interactions over millimeter scales. Elastic processes modifying the particle’s direction of flight are described via multiple scattering theories, and energy losses via stopping powers. In contrast, MCTS codes simulate every single interaction of the primary particle and all secondary electrons down to very low kinetic energies, typically a few electron volts (eV). Simulated interactions are obtained by randomly sampling the corresponding interaction cross sections. Total interaction cross sections define the probability of events like ionization or electronic excitation. Differential cross sections determine the energy and direction of particles after interaction. These cross sections are thus the basic ingredient of any MCTS code.

Over the last four to five decades, many MCTS codes have been developed by various research groups, though not all of them are publicly available [25]. Depending on the main objective of their development, they vary in the type of particles and materials they can simulate. Generally, these codes can simulate electron tracks in liquid water, often used as a surrogate for biological material. Using data for liquid water (as opposed to water vapor) is an important prerequisite for the use of these codes in radiobiology because electron interaction probabilities at low energies depend strongly on the material’s phase (condensation state).

Most codes can also simulate other particles like photons, protons, and ions. Some were developed dedicatedly to supporting experimental nanodosimetry and detector design [26,27]. These codes can simulate tracks of electrons, protons or alpha particles in tissue-equivalent gases such as nitrogen or propane. Others use theoretical models for interaction cross sections of electrons or other charged particles in DNA bases [28–30].

Many of these codes were developed by individual research groups in the last decades of the 20^th^ century and, therefore, are often not publicly available. However, several general-purpose Monte Carlo codes have extended their scope over the past fifteen years by adding track-structure capabilities, albeit often limited to electron transport in liquid water. These additions enable a broader scientific community to use track structure simulations. In particular, this is the case for the code series Geant4 (GEometry ANd Tracking), PHITS (Particle and Heavy Ion Transport code System), and MCNP (Monte Carlo N Particle). Geant4 [31–33] has its Geant4-DNA extension [34–38], also available in derived codes such as TOPAS/TOPAS n-Bio [39,40] and GATE [41]. Similarly, PHITS has been extended by track-structure modes (PHITS-TS) [21,42,43]. A track-structure module is also available in MCNP; however, its low-energy cross section models are limited to atomic targets and may differ significantly due to not considering molecular and other low-energy physics [44].

While most MCTS codes can simulate electron tracks in liquid water, the cross sections employed may differ between codes. This is because different theoretical models were derived from the few experimental data available [45,46]. Therefore, simulations of microdosimetric and nanodosimetric quantities characterizing track structure can yield very different results depending on the MCTS code used [47–49].

All the underlying cross section models have been validated as far as possible by comparing integral quantities such as stopping powers or particle ranges [50,51]. Since direct measurement of microdosimetric or nanodosimetric quantities (e.g., lineal energy or ionization clusters) in water with present technology is a challenge still to be mastered, the benchmarking of the simulation results with experimental data is hampered. The use of gas detectors for this purpose relies on the use of liquid water cross sections for the necessary material-density scaling procedure [52]. The resulting experimental uncertainties are high [53]. Therefore, assessing the systematic uncertainty of the results obtained with MCTS codes is difficult.

Working group 6 (WG 6) of the European Radiation Dosimetry Group (EURADOS e.V.) is concerned with the quality assurance of computational radiation dosimetry methods [54]. Within WG6, Task Group 6.2 is dedicated to micro- and nanodosimetry. The present work is part of this group’s activity to study the variability of results for nanodosimetric quantities obtained with different codes. The goal is to identify how much of this variation comes from differences in the cross section datasets used in the codes to simulate low-energy electron transport. Low-energy electrons are of particular interest because they play a central role in energy deposition and biological damage. For example, electrons below 5 keV contribute about one-third of the energy imparted by ^60^Co gamma-rays and nearly half from 220 kVp X-rays. These low-energy electrons are mainly responsible for the induction of DNA double-strand breaks [55].

**Fig 1:**
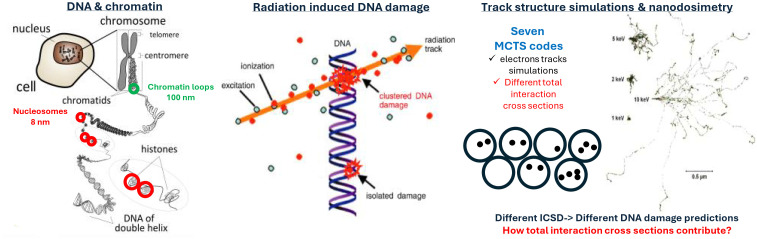
Illustration of the motivation and objective of this work. Left: DNA and chromatin exhibit hierarchical organization, from the DNA double helix to nucleosomes, chromatin loops, and chromosomes within the nucleus (modified from https://commons.wikimedia.org/wiki/File:Chromosomeen.svg, Creative Commons Attribution 3.0 Unported license). Middle: Ionizing radiation produces clustered energy depositions—mainly via secondary low-energy electrons—that lead to complex DNA damage such as double-strand breaks. Right: Nanodosimetry studies these processes using experiments and Monte Carlo track-structure (MCTS) simulations. Differences in the interaction cross sections assumed by various MCTS codes result in distinct ionization cluster patterns and contribute to uncertainty in modeling radiation-induced DNA damage.

The present work and its motivation are schematically illustrated in Fig 1. This paper focuses on the code-dependence of simulated frequency distributions of the number of ionizations in a nanometric volume (termed ionization cluster size distribution, ICSD) produced by low energy electrons. Specifically, we tested the hypothesis that differences in total interaction cross section assumptions largely drive inter-code variability in nanodosimetry results. ICSDs were selected mainly because they are highly sensitive to differences in the MTCS codes used. In addition, they can be measured using gas-based detectors [56] and have been linked to biological effects like DNA double-strand breaks and other types of DNA damage, eventually driving cell fate [15]. However, further efforts in benchmarking ICSD results or exploring their link to radiobiology are beyond the scope of this work. Of note, the few experimental measurements of ICSDs with monoenergetic electrons were performed with a nanodosimeter for which the ion detection efficiency was not determined independently. Instead, it was determined by matching results of MCTS simulations of electrons in nitrogen gas to the measured data [57,58]. Following the same approach, a benchmark of the ICSDs obtained from the MTCS codes included in the present work would require a determination of the nanodosimeter efficiency and different efficiencies would be obtained for each code. Overall, this would not provide additional information on the key question of the investigation, namely, how much of the differences between different MTCS codes are due to different total interaction cross sections used in these codes.

To investigate how much simulated ICSDs (and nanodosimetric quantities derived from them) vary across codes, tracks of electrons with energies from 20 eV to 10 keV were simulated in liquid water. This energy range slightly exceeds the biologically most relevant range (<5 keV). Simulations were first run using each code’s original cross sections. Then, a common cross section dataset, derived *ad-hoc* for this work and not based on any theoretical model (see later for details) was introduced into each code, and the simulations were repeated. While this approach is conceptually simple, it requires cooperation from many researchers working with different MCTS codes. This approach is a key novel aspect of this work compared to earlier code intercomparisons in which only the variability of results by codes with their original cross sections were considered [49,59,60]. Beyond an intercomparison study, the present multi-code work used original vs specifically modified codes, using a common total interaction cross section dataset. The obtained quantitative results highlight the uncertainty in nanodosimetric quantities that can be attributed to variations in total interaction cross-section data.

All these investigations were performed using a simplistic simulation geometry to avoid potential misinterpretations of the simulation tasks that can otherwise lead to additional variance between the results of different codes as was found in past intercomparison exercises in computational dosimetry [61–66]. Only the total cross sections for the interaction types involved were changed. Including differential cross sections would have implied more substantial changes to the codes which would go beyond the scope of this study and be associated with a risk of potential errors. Ultimately, this choice allows the two contributions of the models (total and differential cross sections) to be separated, as discussed later in this article.

Finally, as a sensitivity test and further progress from previous intercomparison studies, DNA damage simulations were performed with one of the codes, PARTRAC, using both its original and the common cross section datasets. This provided indicative insights into how variations in cross section data may impact predictions on biologically relevant endpoints.

## Materials and methods

### Participating Monte Carlo track structure codes

To evaluate how cross section differences affect simulation results, it was important to gather a representative set of MCTS codes. EURADOS Task Group 6.2 includes researchers who use various MCTS codes. After publicizing the study, additional experts joined to contribute with their codes, time and expertise.

Although more than 20 MTCS codes exist (cf. Table 1 in [25]), it was unrealistic to include all of them. Still, the codes included represent a wide spectrum of those actively used in nanodosimetry. These include:

**Table 1:** Mean value of the Wasserstein distances between the ICSDs from the original codes with respect to the mean ICSD for each electron energy. The second row shows these values divided by the average M_1_ value for all the original codes (see Table 2), to help compare ICSD differences across electron energies.

Energy (eV)	20	50	100	300	600	1000	5000	10000
**Mean value of the *W***_**1**_ **distances for original codes**	0.19 ± 0.04	0.35 ± 0.08	0.56 ± 0.13	0.66 ± 0.15	0.39 ± 0.09	0.23 ± 0.04	0.70 ± 0.12	0.40 ± 0.01
**Mean value of the *W***_**1**_ **distances divided by mean *M***_**1**_ **value**	0.37 ± 0.10	0.19 ± 0.05	0.15 ± 0.04	0.12 ± 0.03	0.13 ± 0.03	0.12 ± 0.02	0.103 ± 0.018	0.103 ± 0.004

- Geant4-DNA (with the so-called physics models option 2, option 4 and option 6) [34–38],
- PHITS-TS [42,43],
- PARTRAC [18],
- PTra (Physikalisch-Technische Bundesanstalt track structure code) [26,27,52]
- MCwater [67] an inhouse MCTS code from the Ferhat Abbas University Setif 1, Algeria, that uses published models to account for inelastic and elastic interactions of electrons in liquid water [68–71].

The inelastic and elastic interaction cross section models used by each of these codes for simulating electron transport in liquid water are summarized in S1 Table in S1 File, along with the corresponding references.

The Geant4-DNA code appears herein three times with different options. Indeed, Geant4 and its extension Geant4-DNA are not just “off-the-shelf” MC codes but toolkits that enable users to build their customized track structure simulation. In Geant4-DNA, several physics models are available for simulating low-energy electron transport, regarding both inelastic and elastic interactions. The developers have recommended specific sets of models, often referred to as “physics constructors” or “options.” For this study, three such options (options 2, 4, and 6) were included, each with a different interaction cross section dataset. As a result, they are treated as separate codes for the present analysis. A recent study by Kyriakou et al. [72] compares these three options in terms of electron cross sections, stopping powers, and ranges.

It is important to highlight that this article does not aim to judge the accuracy of the cross section models used by different codes. Comprehensive comparisons of those models are available elsewhere (e.g., [73,74]). Herein, the model descriptions are provided only to help identify which codes use similar or dissimilar cross section data.

### Simulated configuration and quantities

ICSDs produced in a nanometric sphere of liquid water by monoenergetic electrons starting at its center were calculated. Monoenergetic electrons were used to clearly identify the dependence of the variations on electron energy. The sphere diameter was 8 nm for electron energies of 20, 50, 100, 300, 600 and 1000 eV, and 100 nm for 5 and 10 keV.

The 8 nm sphere corresponds approximately to the size of a nucleosome, while the 100 nm one reflects chromatin fiber loops. These sizes also facilitate comparisons with previous work [49,59]. The target sphere was immersed in a larger liquid-water ‘world region’, to allow for potential backscattering of the electrons. The size of the world region was not strictly harmonized among the codes, but this did not critically affect the results.

All codes used their track-structure mode with the lowest possible energy cutoff, typically between 1 and 10 eV, depending on the code. Results for each energy were obtained with 100,000 electrons to keep statistical uncertainties well below expected inter-code differences.

From the results on the ICSDs in the 8 or 100-nm diameter spheres sent by the contributors, different nanodosimetric quantities were calculated and compared: the mean number of ionizations (*M*_1_), and the probabilities of obtaining two or more ionizations (*F*_2_) and three or more ionizations (*F*_3_), i.e., the probabilities of obtaining an ionization cluster or a complex ionization cluster [75]. These quantities correspond to the first momentum of the ICSD and to elements of the complementary cumulative ICSDs and were calculated according to Eq 1, in which *P*_*ν*_ denotes the probability for an ionization cluster size *ν* and $\nu_{m}$ the maximum cluster size:


$$\begin{array}{c} F_{k}=\sum {\mathrm{\backslash nolimits}}_{i=k}^{\nu_{m}}P_{\nu }\;;\;M_{k}=\sum {\mathrm{\backslash nolimits}}_{i=k}^{\nu_{m}}\nu^{k}P_{\nu }\;\; \end{array}$$ (1)

Note that the simulations did not include the interaction producing the initial electrons (e.g., photon interactions or radioactive decay) and did not include the ion produced by this interaction. Only interactions occurring during the transport of the low-energy electrons were simulated.

### Modification of the MCTS codes: Common interaction cross sections

The methodology used in this study was straightforward but required the contributors to modify their MCTS codes, a task possible only for those with access to the source code and in-depth knowledge of its structure.

First, the contributors were asked to extract the default interaction cross section data used in their code for interactions of electrons in liquid water. A template was provided to standardize the reporting. The template listed: the electron kinetic energies, the cross sections values for ionization of the 5 water molecular orbitals and the 5 electronic excitations of the water molecule typically considered in these codes, and the total elastic interaction cross section. The energy grid spanned from 8 eV to 1 MeV for electronic excitations and elastic scattering; and from 10 eV upward for ionizations (as the ionization threshold in liquid water slightly exceeds this value). The energy grid contained at least 9 values per decade and up to 50 points per decade in regions of rapid cross sections variations.

With MCTS codes that include the cross section data as look-up tables, the values requested in the template were obtained from the tables by using the interpolation methods implemented in the codes. For MTCS codes that calculate the cross sections directly from analytical formulae, these formulae had to be extracted from the source code to enable the calculation of the values requested for the template.

After compiling the templates filled by the contributors in the initial phase of the project (some others joined afterwards and one withdrew, see below), mean values for the different interaction cross sections were calculated for each electron energy and each ionization and electronic excitation channel. The resulting cross sections are hereafter referred to as “common cross sections”. Specifically,

- for ionization and excitation by electrons of a given kinetic energy, the scattering cross sections were determined as the arithmetic mean of the corresponding values from Geant4-DNA options 2, 4 and 6, PTra, PARTRAC and the relativistic binary-encounter Bethe (BEB) values from PHITS-TS-v3.02;
- for elastic scattering, the average values were the arithmetic mean of the data from the models used by default in these six codes and additionally the values from the ELSEPA model (also available as option in Geant4-DNA).

The MCwater code and the version 3.34 of PHITS-TS joined the project after common cross sections were established. To avoid double work for the other codes, the already established common cross section data set was maintained. Therefore, the original cross sections from these two codes are not considered in the common cross section data set.

Several points are important to note with respect to this methodology. First, all codes and their cross section models were independently and previously benchmarked against integral data as mentioned above. Second, the cross section data used in the codes (as tabulated values or formulae) contain no information on uncertainties associated with them. Therefore, there is no *a priori* rationale for considering cross sections used in any MTCS code to be more accurate than the others. Similarly, there are no experimental cross section data that could be used as reference. There is also no commonly accepted ground-truth theoretical data for this purpose. Furthermore, since the goal was to evaluate the variability and not the accuracy of the codes, the simple averaging of the values from all codes is the best heuristic way to construct a reference for comparing them and measuring their variability. For the same reason, it was not deemed necessary to re-evaluate these common cross sections when two new codes or code versions were added to the exercise. It must be stressed that the common, averaged cross section database is not intended to provide a realistic physical model of low-energy electron interactions in water, nor is it based on any theoretical assumption. It served merely as an unbiased mean allowing the resulting variations to be interpreted as an uncertainty estimate. Although based on seven codes only, this approach provides a representative basis for the analysis. Based on all these considerations, to avoid its potential misuse, the averaged dataset is not provided in numerical form.

The contributors then implemented the common cross sections in their codes (in a tabular form) and re-ran the ICSD simulations. The total ionization, electronic excitation and elastic cross sections originally used by the participating MCTS and the corresponding common-set of cross sections are shown in S1-S3 Figs in S1 File.

Since the objective was to isolate the influence of total interaction cross sections on the variability of results between codes, the rest of the parameters involved in the simulations were kept constant. This means that out of the basic ingredients of MCTS simulations illustrated in Supplementary S4 Fig in S1 File, only the total cross sections were harmonized, while the other ingredients were allowed to vary among the codes. More precisely:

- The ratio of the cross sections differential in scattering angle to the total cross section of the process were kept as in the original MCTS code. This means that:
- For elastic scattering, only the interaction probability for a given electron energy was prescribed by the common cross section dataset. The electron’s direction of motion after the elastic collision was sampled as done originally in the given MCTS code. Yet, the influence of differential cross sections has been studied exemplarily for the three versions of Geant4-DNA.
- For electronic excitation, also only the interaction probability was harmonized, while the angular distribution of the scattered electron’s direction was sampled as in the original code.
- For ionizations, the interaction probability was harmonized, whereas the angular distributions of both the projectile and the target electrons’ directions of motion were sampled as in the original code.
- Cross sections for ionizing interactions differential in energy were not modified. The probability of a given kinetic energy for the secondary electron after the ionization of a given shell (and thus for the projectile, as they are related via energy and momentum conservation) was kept as in the original codes.
- Binding energies for ionization shells and excitation shells were also kept as in the original codes.
- The specific interpolation methods and/or algorithms (such as linear vs log-log interpolation or the energy grid) used in the different MCTS to calculate the interaction cross sections from the data tables were kept unmodified (see S1 Table in S1 File).

Therefore, any residual variability among the modified MCTS will reflect the contribution of those unchanged elements. In other words, the comparison between original and total cross section-harmonized MCTS provides an empirical measure of the importance of differential cross sections and implementation differences to nanodosimetric variability.

### Quantitative assessment of the differences between ICSDs

Inter-code variability was assessed for the distributions and for the nanodosimetric parameters *M*_1_, *F*_2_, and *F*_3_ derived from the ICSDs. The total and relative standard deviation were used as metrics for the variability of these quantities and were determined between the results obtained by different contributors with either the original or the common cross section datasets.

For quantifying differences between ICSDs, the Wasserstein distance *W*_1_ [76] was used, defined for two discrete distributions as:


$$\begin{array}{c} W_{\text{1}}=\sum {\mathrm{\backslash nolimits}}_{i}^{bins}\left|\delta_{i}\right|\text{ with }\left\{ \begin{array}{c} \delta_{\text{0}}=\text{0}\\ \delta_{i+\text{1}}=\delta_{i}+x_{i}-y_{i} \end{array}\right.\;\; \end{array}$$ (2)

Here, $x_{i}$ and $y_{i}$ represent the probabilities in the *i-th* bin from two different distributions—either from two different codes or from the same code using original vs. common cross sections. Further, $\delta_{i+\text{1}}$ is the difference between the cumulative distributions in the *i-th* bin. This metric is also called the Earth-Mover distance. It can be interpreted as the minimum energy cost (in physical terms: work) of moving and transforming bin by bin (pile by pile) one probability distribution into the shape of the other distribution. For example, if two normalized distributions (such as ICSDs here) are identical except for a rigid shift, their *W*_1_ equals the number of bins by which they are offset. Therefore, the higher *W*_1_, the higher is the difference between the compared distributions for the same number of bins taken into account.

In previous work [59], the variability of microdosimetric and nanodosimetric distributions was analyzed bin-by-bin, and Pearson’s correlation coefficients were calculated for each pair of datasets from different contributors. However, that method cannot be applied here, since this study compares each result from a code to an average over the results of all contributors for each ionization cluster size, i.e., a value correlated with each of the datasets from which it was constructed. For the same reason, the χ^2^ metrics is also not directly applicable. Therefore, only a comparison of magnitude between the variation across all codes and the statistical uncertainties of the quantities obtained with the different codes is considered here.

In this work, the Wasserstein distances were used to quantify three types of deviations:

a/ the “initial variability” that corresponds to the variability among original MCTS codes by comparing each original ICSD to a mean ICSD calculated with all of them;
b/ the “final variability” of the modified MCTS codes by comparing the ICSD of each modified code to the mean ICSD calculated with all the modified codes;
c/ for each code the deviation of the ICSDs obtained with the original code and its modified version.

The initial variability between codes results from all the factors contributing to the calculation of ICSDs. On the other hand, the final variability reflects differences excluding total cross sections. Therefore, the reduction in variability quantifies how much of the variation is specifically due to the total interaction cross sections.

ICSD distributions hugely vary with electron energy, both qualitatively and quantitatively. In particular, the mean numbers of ionizations M_1_ vary. Absolute values of W_1_ distances should therefore not be compared among diverse electron energies. To address this issue, also W_1_/M_1_ are reported. These ratios indicate how big is the difference among two distributions relative to their mean, thus providing values that facilitate such comparisons.

### Uncertainty assessment and statistical analysis

For each ICSD, the uncertainties of the probabilities *P*_*ν*_ of ionization cluster size *ν* and of the cumulative probabilities *F*_2_ and *F*_3_ were determined using binomial sampling statistics according to Eq 3, in which *x* stands for the corresponding probability value and *N* for the number of simulated primary particles.


$$u(x)=\sqrt{\frac{x(\text{1}-x)}{N}}$$ (3)

The uncertainty of *M*_1_ was determined following the law of error propagation according to the GUM (guide to the expression of uncertainties) [77] according to Eq 4:


$$u(M_{\text{1}})=\sqrt{\frac{M_{\text{2}}-{M_{\text{1}}}^{\text{2}}}{N}}$$ (4)

To quantify the variability of the nanodosimetric parameters obtained by each code, the mean value (MV) over all codes, the standard deviation (SD) and relative standard deviation (RSD) were determined, where the latter is the ratio of the SD to the MV. The uncertainties of the SD and RSD were estimated according to the GUM [77], which leads to the expressions given in Eqs 5 and 6, where *n*_*c*_ denotes the number of codes, *x*_*k*_ the result obtained with code *k*, and *u*(*x*_*k*_) the uncertainty of *x*_*k*_ according to Eqs 3 or 4:


$$\begin{array}{c} u(\text{SD})=\frac{\text{1}}{n_{c}\times \text{SD}}\sqrt{\sum {\mathrm{\backslash nolimits}}_{k=\text{1}}^{n_{c}}{\left(x_{k}-\text{MV}\right)}^{\text{2}}\times {u(x_{k})}^{\text{2}}} \end{array}$$ (5)

$$\begin{array}{c} u(\text{RSD})=\frac{\text{1}}{n_{c}\times \text{RSD}\times \text{MV}}\sqrt{\sum {\mathrm{\backslash nolimits}}_{k=\text{1}}^{n_{c}}{\left(\frac{x_{k}}{\text{MV}}-\text{1}-{\text{RSD}}^{\text{2}}\right)}^{\text{2}}\times {u(x_{k})}^{\text{2}}} \end{array}$$ (6)

The uncertainty of the MV was determined by combining the uncertainty estimate from error propagation with the standard error from a statistical estimate of the uncertainty of the mean applying Eq 7:


$$\begin{array}{c} u(\text{MV})=\sqrt{\frac{\text{1}}{n_{c}}\sum {\mathrm{\backslash nolimits}}_{k=\text{1}}^{n_{c}}{u(x_{k})}^{\text{2}}+\frac{\text{1}}{n_{c}-\text{1}}{\text{SD}}^{\text{2}}} \end{array}$$ (7)

The uncertainty of the Wasserstein distance *W*_1_ was obtained by uncertainty propagation assuming statistical independence of the two distributions. When comparing an ICSD from a code with the average across all codes, this assumption neglects the existing correlation between the two distributions. The resulting bias leads to an overestimation of the uncertainty. This also applies to the uncertainties of average Wasserstein distances (across all codes). However, this overestimation would only be relevant if the variations were comparable to or smaller than these uncertainties, which is not the case.

### Potential impact of cross section differences on predicted DNA damage

To illustrate how the use of different interaction cross section data may affect predicted biological effects, simulations of DNA damage were performed using the PARTRAC code, with both the original and common cross section datasets.

The simulation setup comprised a spherical lymphocyte cell nucleus (10 µm in diameter) incorporating a multi-scale chromatin model. A 2.11 µm liquid water shell surrounded the nucleus to include potential re-entering of backscattered electrons into the nucleus, as in previous work [78]. The chromatin model included an atomic description of the DNA, its winding around nucleosomes, formation of chromatin fiber, its loops, spherical chromatin domains and chromosomes in human interphase nuclei [19]; the total DNA content was 6.6 Gbp. Monoenergetic 100 eV or 10 keV electrons were initiated isotropically from homogeneously distributed random points within the nucleus. Both direct damage (from electron interactions with DNA) and indirect effects (from water radiolysis products) were considered, using standard assumptions and parameter values [19,78].

For 100 eV and 10 keV electrons, respectively, in total 10^7^ and 10^6^ tracks were simulated in 100 runs (with 10^5^ or 10^4^ tracks per run). These particle numbers were chosen based on previous similar simulations to provide sufficient statistics within reasonable time (several hours CPU on a mid-class multi-thread laptop). The following classes of DNA damage were scored [78]: single-strand breaks (SSBs); double-strand breaks (DSBs, defined as strand breaks on opposite strands within 10 bp); DSB clusters (containing at least 2 DSBs within 25 bp); and DSB sites (not discriminating between an isolated DSB and a DSB cluster but scoring both as a single DSB site).

It is important to note that the DNA damage simulations were performed solely to illustrate the impact of cross section variations on the biologically relevant predictions, i.e., as a sensitivity test. Their results should not be interpreted as a confirmation of the DSB modelling but highlight that the outcomes depend on the assumptions about the underpinning cross sections.

## Results

### Variability in ICSDs from different MCTS codes

**Fig 2:**
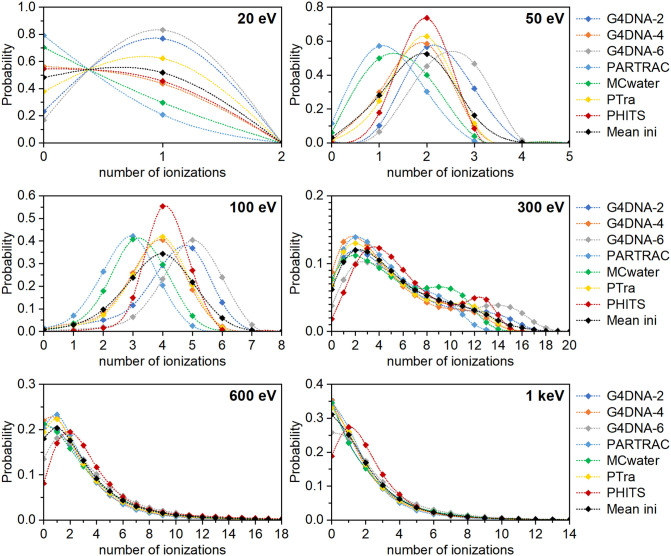
ICSDs produced in a liquid water sphere of 8 nm in diameter by a point source of mono-energetic electrons in its center. The initial electron energies are indicated in the graphs. Each color represents a different MCTS code using its original interaction cross sections. The black curve shows the mean ICSD (“Mean ini”) across all codes. Note: the lines are only there to guide the eye, as non-integer values for the number of ionizations are meaningless.

**Fig 3:**
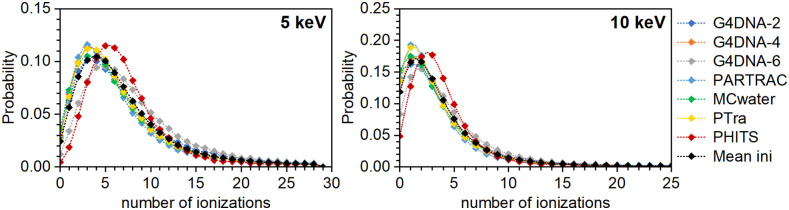
ICSDs produced in a liquid water sphere of 100 nm in diameter by a point source of mono-energetic electrons at the energies indicated in the graphs. The data were obtained by the diverse MCTS codes using their original interaction cross sections. The black symbols indicate the mean ICSD (“Mean ini”) across all codes at the given electron energy. Note: the lines are only there to guide the eye, as non-integer values for the number of ionizations are meaningless.

Fig 2 shows the ICSDs obtained for low-energy electrons in an 8 nm diameter liquid water sphere. Fig 3 displays the results for a 100 nm diameter water sphere. Numerical data are listed in S2 Table in S1 File. To simplify the notation, the results obtained with the three Geant4-DNA options are labelled as G4DNA-2, G4DNA-4 and G4DNA-6 in the figures and tables.

Differences between these curves are evident; they are especially pronounced at low electron energies (i.e., below 300 eV) and for low ionization numbers in the volume. These differences correlate with those in the original cross sections used in the codes (see S1-S3 Figs in S1 File). For instance, PARTRAC uses notably smaller ionization cross sections at the lowest electron energies than the other involved MCTS codes (S1 Fig in S1 File). Consequently, it predicts that about 80% of 20 eV electrons induce no ionization, while 20% cause one ionization in the 8 nm sphere. Geant4-DNA options 2 and 6 yield nearly the opposite distributions (Fig 2). At higher energies, this relationship between cross sections and ICSDs is less evident.

In Figs 2 and 3, the black symbols indicate the mean value of all datapoints for the same ionization cluster size, i.e., the mean ICSD across the participating codes in their original version. This “mean initial” ICSD served as the reference to calculate the Wasserstein (*W*_1_) distances for each ICSD. The resulting *W*_1_ distances are listed in S3 Table in S1 File. The mean value of all the calculated *W*_1_ distances at a given electron energy, given in the first row of Table 1, is a measure of the variability among the original codes.

These absolute Wasserstein distance values should not be overinterpreted in absolute terms or directly compared across different electron energies. This is because the number of bins in the ICSD histograms varies with energy. A greater number of bins naturally increases the potential magnitude of the *W*_1_ distance. To allow meaningful comparisons across energies, the *W*_1_ distances were normalized by dividing them by the average value of *M*_1_ across all codes at each energy (values shown in the third column from the right in Table 2). The resulting relative *W*_1_ distances per ionization in the target volume, reported in the second row of Table 1, offer a better measure for comparing the differences of the ICSD histograms across electron energies. In line with the trends visible in Fig 2, the *W*_1_/*M*_1_ ratios indicate that the relative ICSD variability among the codes is highest at the lowest electron energies.

**Table 2:** Mean numbers of ionizations (M_1_) in liquid water spheres of 8 nm diameter (for 20 eV – 1 keV electron energies) and 100 nm diameter (for 5 and 10 keV electron energies), respectively, obtained with the seven original MCTS codes. The mean value (MV) and standard deviation (SD) across all codes and the resulting relative standard deviation (RSD) for each electron energy are also given to quantify the variability of results.

Energy (eV)	G4DNA-2	G4DNA −4	G4DNA- 6	PARTRAC	MCwater	PTra	PHITS	MV	SD	RSD
**20**	0.7680 ± 0.0013	0.4370 ± 0.0016	0.8330 ± 0.0012	0.2080 ± 0.0013	0.2970 ± 0.0014	0.6220 ± 0.0015	0.4550 ± 0.0016	0.52 ± 0.10	0.2338 ± 0.0010	0.4521 ± 0.0010
**50**	2.206 ± 0.002	1.771 ± 0.002	2.429 ± 0.002	1.212 ± 0.002	1.419 ± 0.002	1.8426 ± 0.0020	1.9027 ± 0.0016	1.83 ± 0.17	0.4202 ± 0.0010	0.2301 ± 0.0004
**100**	4.303 ± 0.004	3.648 ± 0.004	4.829 ± 0.003	2.808 ± 0.003	3.167 ± 0.003	3.723 ± 0.003	4.093 ± 0.002	3.8 ± 0.3	0.6846 ± 0.0012	0.1804 ± 0.0003
**300**	5.344 ± 0.013	4.545 ± 0.011	6.655 ± 0.015	4.437 ± 0.010	5.215 ± 0.012	5.015 ± 0.012	6.040 ± 0.012	5.3 ± 0.3	0.794 ± 0.005	0.1493 ± 0.0008
**600**	2.905 ± 0.010	2.541 ± 0.009	3.587 ± 0.011	2.498 ± 0.008	2.989 ± 0.010	2.768 ± 0.009	3.759 ± 0.011	3.0 ± 0.2	0.491 ± 0.004	0.1633 ± 0.0012
**1000**	1.826 ± 0.007	1.668 ± 0.007	2.261 ± 0.008	1.572 ± 0.006	1.873 ± 0.007	1.761 ± 0.007	2.198 ± 0.007	1.88 ± 0.11	0.259 ± 0.003	0.1380 ± 0.0013
**5000**	7.119 ± 0.017	6.575 ± 0.016	8.112 ± 0.017	6.470 ± 0.016	6.500 ± 0.016	6.529 ± 0.016	7.304 ± 0.013	6.9 ± 0.3	0.613 ± 0.006	0.0883 ± 0.0008
**10000**	4.024 ± 0.014	3.976 ± 0.013	4.532 ± 0.014	3.606 ± 0.013	3.709 ± 0.013	3.593 ± 0.012	4.063 ± 0.011	3.93 ± 0.14	0.331 ± 0.005	0.0843 ± 0.0012

### Variability in the nanodosimetric quantities from the original MCTS simulations

Table 2 and Table 3 give the values of the nanodosimetric quantities *M*_1_ and *F*_2_, respectively, obtained from the original MCTS code simulations.

**Table 3:** Values of the probability of obtaining more than 2 ionizations (F_2_) in liquid water spheres of 8 nm diameter (for 50 eV-1 keV electron energies) and 100 nm diameter (for 5 and 10 keV electron energies), respectively, obtained from the ICSDs calculated with the original MCTS codes. The mean value (MV) and standard deviation (SD) across all codes and the resulting relative standard deviation (RSD) for each electron energy are also given to quantify the variability of results. There is no row for 20 eV as this energy is sufficient for producing up to a single ionization only, so that F_2 _≡ 0.

Energy (eV)	G4DNA-2	G4DNA-4	G4DNA-6	PARTRAC	MCwater	PTra	PHITS	MV	SD	RSD
**50**	0.8893 ± 0.0010	0.6859 ± 0.0015	0.9332 ± 0.0008	0.3140 ± 0.0015	0.4401 ± 0.0016	0.7408 ± 0.0014	0.8202 ± 0.0012	0.69 ± 0.09	0.2317 ± 0.0010	0.3363 ± 0.0008
**100**	0.9621 ± 0.0006	0.9506 ± 0.0007	0.9886 ± 0.0003	0.9151 ± 0.0009	0.9549 ± 0.0007	0.9633 ± 0.0006	0.9961 ± 0.0002	0.962 ± 0.011	0.0266 ± 0.0010	0.0277 ± 0.0002
**300**	0.8140 ± 0.0012	0.7807 ± 0.0013	0.8861 ± 0.0010	0.8124 ± 0.0012	0.8184 ± 0.0012	0.8154 ± 0.0012	0.9245 ± 0.0008	0.84 ± 0.02	0.0503 ± 0.0010	0.0602 ± 0.0004
**600**	0.5900 ± 0.0016	0.5550 ± 0.0016	0.6840 ± 0.0015	0.5650 ± 0.0016	0.5920 ± 0.0016	0.5830 ± 0.0016	0.7494 ± 0.0014	0.62 ± 0.03	0.0720 ± 0.0010	0.1167 ± 0.0008
**1000**	0.4170 ± 0.0016	0.3930 ± 0.0015	0.5020 ± 0.0016	0.3810 ± 0.0015	0.4280 ± 0.0016	0.4090 ± 0.0016	0.5380 ± 0.0016	0.44 ± 0.02	0.0588 ± 0.0010	0.1342 ± 0.0013
**5000**	0.9033 ± 0.0009	0.9016 ± 0.0009	0.9541 ± 0.0007	0.9018 ± 0.0009	0.8906 ± 0.0010	0.9082 ± 0.0009	0.9768 ± 0.0005	0.919 ± 0.013	0.0325 ± 0.0010	0.0353 ± 0.0003
**10000**	0.6880 ± 0.0015	0.7100 ± 0.0014	0.7745 ± 0.0013	0.6660 ± 0.0015	0.6720 ± 0.0015	0.6750 ± 0.0015	0.8246 ± 0.0012	0.72 ± 0.02	0.0608 ± 0.0010	0.0849 ± 0.0007

The relative standard deviation (RSD) among the codes allows comparing the variability in simulation results between different electron energies. In the case of *M*_1_, the variability increases with decreasing electron energies. At 20 eV, where only 0 or 1 ionizations are possible, the RSD is as high as 45%. As *M*_1_ is the mean value of the ICSD, the values in Table 2 can be understood from the data shown in Figs 2 and 3. For instance, at 100 eV, PARTRAC and Geant4-DNA option 6 yield similar-shaped ICSDs, but one is shifted by 2 ionizations compared to the other. Hence, also the *M*_1_ values differ by about two (*M*_1_ = 2.808 ± 0.003 or 4.829 ± 0.003, respectively).

The RSD of the *F*_2_ values is also high at low energy (34% at 50 eV). However, it decreases dramatically at 100 eV. This is because nearly all codes predict similar probabilities *P*_0_ and *P*_1_ of 0 and 1 ionizations, respectively, at this energy, and *F*_2_ = 1-*P*_0_-*P*_1_. Despite this similarity in *F*_2_, the ICSDs at 100 eV differ considerably between codes (Fig 2). This illustrates that *F*_2_ alone does not capture the full differences between ICSDs. For higher electron energies (300–1000 eV), the RSD of the *F*_2_ values increases and then decreases again at 5 and 10 keV. The latter is likely due to the larger volume, which smooths out differences.

The same trend is seen in the values of *F*_3_ (see S6 Table in S1 File). At 50 eV, the RSD is 104%. At 100 eV, it decreases to 13%. In this case, it is the sum of *P*_0_, *P*_1_ and *P*_2_ that determines *F*_3_ so that differences at low ionization numbers heavily affect its value.

### Variability in ICSDs from MCTS codes with common cross sections

**Fig 4:**
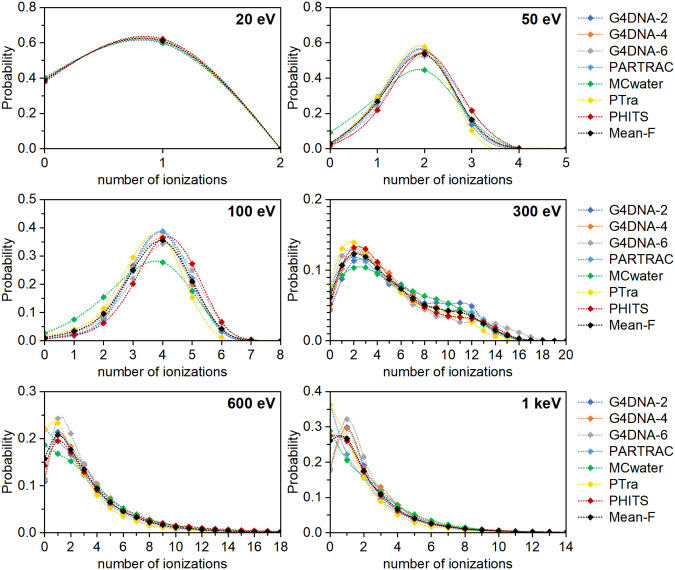
ICSDs in spherical targets of 8 nm diameter predicted by diverse MCTS codes using the common cross section dataset. The labels indicate initial electron energies. The data shown in black are the ICSDs (“Mean-F”) averaged across all the modified codes. Note: the lines are only there to guide the eye, as non-integer values for the number of ionizations are meaningless.

**Fig 5:**
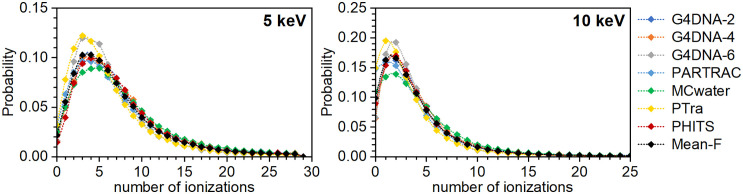
ICSDs obtained after modifying the MCTS codes with common cross sections for monoenergetic electrons of 5 and 10 keV in 100 nm diameter spherical targets. The data shown in black are the ICSDs (“Mean-F”) averaged across all the modified codes. Note: the lines are only there to guide the eye, as non-integer values for the number of ionizations are meaningless.

The results of the ICSDs for monoenergetic electrons from 20 eV to 1 keV in spherical targets of 8 nm diameter determined with MCTS codes using the common interaction cross sections are shown in Fig 4. Fig 5 shows the corresponding results for 5 and 10 keV electrons in larger target volumes of 100 nm diameter. As before, the different colors correspond to the different codes, and the black symbols in each figure indicate the average (“Mean-F”) across all modified codes at each energy.

A comparison of Fig 4 and Fig 5 with Fig 2 and Fig 3 shows that the use of the common cross section dataset by all MCTS codes hugely reduced the variability of their predictions, in particular for low energy electrons. To quantify the residual variability of the results using the common cross sections, the corresponding Wasserstein distances were calculated for each ICSD obtained with the modified codes to the mean ones shown in black color in Fig 4 and Fig 5. The results can be found in S4 Table in S1 File. Table 4 shows the mean values of these Wasserstein distances between the results of all modified codes and the mean distribution. As with the codes in their original version, the normalized values divided by the average value of the *M*_1_ for the modified codes are also shown in the second line of Table 4. As expected, the resulting values are lower than the ones obtained with original MCTS codes in Table 1. For 20 eV electrons, for which only 0 or 1 ionizations are possible in the target volume, the reduction is by more than a factor of 20. Non-unified MCTS ingredients such as the angular distribution of the secondary electrons play only a minor role at this energy, as the target volume is relatively large compared with the range of 20 eV electrons. Using common total interaction cross sections thus largely reduces the variability among the codes in this case.

**Table 4:** Mean value of the Wasserstein distance between the ICSDs from the modified codes and the mean ICSD for each electron energy. The second row shows these values divided by the average M_1_ value for all the modified codes.

Energy (eV)	20	50	100	300	600	1000	5000	10000
**Mean value of the *W***_**1**_ **distances for the modified codes**	0.007 ± 0.003	0.08 ± 0.02	0.18 ± 0.05	0.32 ± 0.08	0.37 ± 0.06	0.26 ± 0.05	0.62 ± 0.15	0.40 ± 0.11
**Mean value of the *W***_**1**_ **distance divided by mean *M***_**1**_ **value of modified codes**	0.011 ± 0.005	0.045 ± 0.011	0.049 ± 0.012	0.061 ± 0.015	0.12 ± 0.02	0.13 ± 0.02	0.09 ± 0.02	0.10 ± 0.03

At 50, 100 and 300 eV, the inter-code variability is also largely reduced, with the mean Wasserstein distances between the results of all modified codes and the mean distribution decreasing approximately by factors of 4, 3 and 2, respectively. However, at 600 eV and above, the benefit from using a common dataset decreases. The mean value of the *W*_1_ distances increases and is about as high as that obtained with the original codes. This reflects the gradually increasing role of factors other than the total interaction cross sections, in particular differential cross sections (that govern the emission angles and energies of particles post interaction) start to dominate the variability. These were not standardized and remained specific to each MCTS code.

### Variability in nanodosimetric quantities between MCTS codes with common-set cross sections

Table 5 and Table 6 present the nanodosimetric quantities *M*_1_ and *F*_2_, respectively, obtained with the individual MCTS codes using the common cross section datasets. Both tables also include the SD and RSD of these values for each electron energy. The RSD of the *M*_1_ and *F*_2_ values for 20 eV and 50 eV electrons decreased to very low values (from 0.45 with original cross sections to 0.01with the common dataset at 20 eV and from 0.23 to 0.05 at 50 eV). This improvement reflects the reduced variability of ICSDs predicted by individual codes upon using the common cross section dataset (Fig 4 and Fig 5). A similar decrease in the RSD of *M*_1_ value with respect to the initial one shown in Table 2 is observed for 100 eV and 300 eV. However, for electron energies of 600 eV and higher, the RSDs of *M*_1_ with the modified MCTS codes show values similar to those with the original codes. This again points to increasing influence of non-unified code components, such as angular distributions and energy spectra of secondary particles.

**Table 5:** M_1_ values of the ICSDs calculated with the modified MCTS codes. The mean value (MV) and standard deviation (SD) across all codes and the resulting relative standard deviation (RSD) for each electron energy are also given to quantify the variability of results.

Energy (eV)	G4DNA-2	G4DNA-4	G4DNA-6	PARTRAC	MCwater	PTra	PHITS	MV	SD	RSD
**20**	0.5975 ± 0.0016	0.6123 ± 0.0015	0.6088 ± 0.0015	0.6208 ± 0.0015	0.6035 ± 0.0015	0.6085 ± 0.0015	0.6223 ± 0.0015	0.611 ± 0.004	0.0089 ± 0.0005	0.0145 ± 0.0009
**50**	1.824 ± 0.002	1.836 ± 0.002	1.936 ± 0.002	1.857 ± 0.002	1.696 ± 0.003	1.760 ± 0.002	1.973 ± 0.002	1.84 ± 0.04	0.0953 ± 0.0009	0.0518 ± 0.0005
**100**	3.751 ± 0.003	3.701 ± 0.004	3.882 ± 0.004	3.826 ± 0.003	3.376 ± 0.004	3.473 ± 0.004	4.002 ± 0.004	3.72 ± 0.09	0.2224 ± 0.0014	0.0599 ± 0.0004
**300**	5.710 ± 0.012	5.258 ± 0.012	5.235 ± 0.013	5.257 ± 0.012	5.465 ± 0.012	4.577 ± 0.011	5.150 ± 0.012	5.24 ± 0.14	0.346 ± 0.004	0.0661 ± 0.0008
**600**	3.374 ± 0.010	3.234 ± 0.009	2.841 ± 0.008	2.856 ± 0.010	3.376 ± 0.011	2.503 ± 0.009	3.641 ± 0.012	3.12 ± 0.16	0.396 ± 0.004	0.1271 ± 0.0011
**1000**	2.308 ± 0.008	2.290 ± 0.007	2.040 ± 0.007	1.793 ± 0.007	2.288 ± 0.008	1.575 ± 0.006	2.111 ± 0.008	2.06 ± 0.12	0.281 ± 0.002	0.1368 ± 0.0012
**5000**	7.208 ± 0.016	7.285 ± 0.016	6.364 ± 0.014	7.103 ± 0.017	7.614 ± 0.017	6.073 ± 0.015	7.316 ± 0.015	7.0 ± 0.2	0.559 ± 0.005	0.0800 ± 0.0008
**10000**	4.344 ± 0.013	4.352 ± 0.013	3.908 ± 0.012	4.045 ± 0.014	4.587 ± 0.014	3.402 ± 0.012	4.143 ± 0.013	4.11 ± 0.16	0.385 ± 0.004	0.0936 ± 0.0011

**Table 6:** Cumulated probability F_2_ of obtaining 2 or more ionizations in the target volume calculated with the modified MCTS codes. The mean value (MV) and standard deviation (SD) and its relative value (RSD) for each electron energy are also given to quantify the variability of results.

Energy (eV)	G4DNA-2	G4DNA-4	G4DNA-6	PARTRAC	MCwater	PTra	PHITS	MV	SD	RSD
**50**	0.7021 ± 0.0014	0.6982 ± 0.0015	0.7398 ± 0.0014	0.7186 ± 0.0014	0.6152 ± 0.0015	0.6809 ± 0.0015	0.7657 ± 0.0013	0.703 ± 0.020	0.0479 ± 0.0005	0.0681 ± 0.0007
**100**	0.9703 ± 0.0005	0.9614 ± 0.0006	0.9630 ± 0.0006	0.9760 ± 0.0005	0.8985 ± 0.0010	0.9449 ± 0.0007	0.9729 ± 0.0005	0.955 ± 0.011	0.0271 ± 0.0003	0.0283 ± 0.0003
**300**	0.8688 ± 0.0011	0.8528 ± 0.0011	0.8138 ± 0.0012	0.8151 ± 0.0012	0.8350 ± 0.0012	0.7911 ± 0.0013	0.8410 ± 0.0012	0.831 ± 0.011	0.0263 ± 0.0004	0.0317 ± 0.0005
**600**	0.6780 ± 0.0015	0.6779 ± 0.0015	0.6447 ± 0.0015	0.5846 ± 0.0016	0.6454 ± 0.0015	0.5486 ± 0.0016	0.6621 ± 0.0015	0.63 ± 0.02	0.0494 ± 0.0005	0.0778 ± 0.0009
**1000**	0.5238 ± 0.0016	0.5262 ± 0.0016	0.4966 ± 0.0016	0.4155 ± 0.0016	0.5058 ± 0.0016	0.3735 ± 0.0015	0.4654 ± 0.0016	0.47 ± 0.02	0.0582 ± 0.0005	0.1231 ± 0.0012
**5000**	0.9329 ± 0.0008	0.9345 ± 0.0008	0.9278 ± 0.0008	0.9059 ± 0.0009	0.9246 ± 0.0008	0.8902 ± 0.0010	0.9459 ± 0.0007	0.923 ± 0.008	0.0189 ± 0.0003	0.0205 ± 0.0003
**10000**	0.7706 ± 0.0013	0.7764 ± 0.0013	0.7615 ± 0.0013	0.6934 ± 0.0015	0.7568 ± 0.0014	0.6554 ± 0.0015	0.7565 ± 0.0014	0.739 ± 0.019	0.0458 ± 0.0005	0.0620 ± 0.0007

Concerning *F*_2_ values, the decrease in the RSD upon assuming the common cross section dataset (Figs 4 and 5) is pronounced at 50 eV (from 0.3363 ± 0.0008 to 0.0681 ± 0.0007). For other energies, the effect is limited, as the RSDs were already very low with the original MCTS codes (Table 3). This indicates that, for most energies, the *F*_2_ value is relatively insensitive to differences in cross section data—except at the lowest energies where clustering is dominated by rare ionization events. The same trend holds for the *F*_3_ values (S6 and S7 Tables in S1 File ) where the RSD at 50 eV decreases dramatically from 1.041 ± 0.004 to 0.2472 ± 0.0014 upon using common cross sections but remains almost unchanged for the other electron energies.

### Impact of total cross sections on the simulated nanodosimetric results

**Fig 6:**
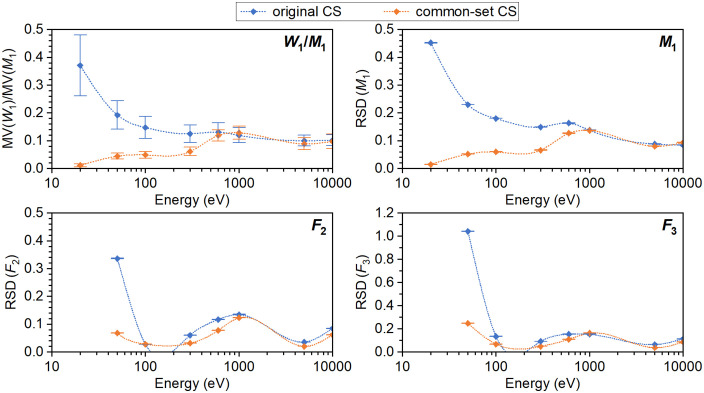
Graphical representation of the ratio of the mean W_1_ to the mean M_1_ values and the relative standard deviations (RSD) for M_1_, F_2_, and F_3_ obtained in this work with original codes and their modified version using common total cross sections. Note: the represented spline curves are only there to guide the eye.

To aid in the interpretation of the results, Fig 6 presents the values obtained in this work representing the inter-code variability, namely the *W*_1_/*M*_1_ values and the RSD for *M*_1_, *F*_2_, and *F*_3_. Comparing these values when using each code’s original cross sections versus the common set highlights the impact of total interaction cross sections on the reduction—or persistence—of inter-code variability.

With the same aim of facilitating the reader’s understanding and compilation of the data obtained, the numerical values are provided in S8 and S9 Tables in S1 File with a summary of the mean values of *M*_1_, *F*_2_ and *F*_3_ obtained with the original codes versus those calculated with the modified codes. S5-S7 Figs in S1 File show a direct comparison of the ICSDs originally obtained with those obtained with the modified MCTS codes.

### Potential impact of cross section differences on predicted initial DNA damage

Table 7 summarizes the results of PARTRAC simulations on energy deposition and DNA damage induction by 100 eV and 10 keV electrons, started isotropically from homogeneously distributed points within the model cell nucleus. Using the original cross section database, the average energy imparted to the nucleus by a 100 eV electron amounted to 99.95 eV, that is, on average only 0.05% of the initial electron energy was deposited outside the nucleus. For 10 keV electrons, on average 8.39 keV were imparted to the nucleus, and about 16% of the initial electron energy was carried away. This energy carried away is due to electrons initiated close to the surface of the nucleus traveling and depositing energy outside the nucleus. Their fraction is negligible for extremely short-ranged 100 eV electrons but notable at 10 keV due to the longer path length (2.77 µm) and penetration depth (1.41 µm) in water. When using the common cross section dataset, these figures changed only marginally to 99.97 eV and 8.33 keV deposited to the nucleus per 100 eV or 10 keV electron, respectively.

**Table 7:** PARTRAC simulations on energy deposition and induction of DNA damage by 100 eV and 10 keV electrons, using the code’s original or the common interaction cross sections. Damage yields are reported as mean values ± standard error of the mean estimated from the 100 runs simulated, with 10^5^ tracks of 100 eV or 10^4^ tracks of 10 keV electrons per run, respectively.

Energy (eV)	Cross section database	Energy deposited within cell nucleus (%)	Absorbed dose in cell nucleus (Gy)	Yields of SSB (Gy^-1^ Gbp^-1^)	Yields of DSB (Gy^-1^ Gbp^-1^)	Yields of DSB clusters (Gy^-1^ Gbp^-1^)
**100**	Original	99.95	305.8	153.8 ± 0.3	5.40 ± 0.05	0.003 ± 0.002
**100**	Common set	99.97	305.9	144.8 ± 0.3	6.20 ± 0.05	0.011 ± 0.002
**10000**	Original	83.9	2650	151.6 ± 0.2	8.87 ± 0.02	0.100 ± 0.002
**10000**	Common set	83.3	2550	147.8 ± 0.2	8.92 ± 0.02	0.107 ± 0.002

To achieve good statistics for the yields of DNA lesions, a total of ten million tracks were simulated for 100 eV electrons and one million of tracks for 10 keV electrons, sub-divided into 100 runs each. The corresponding doses were about 305.8 and 305.9 Gy (about 3.1 Gy per run) for 100 eV electrons using the original and modified cross sections, respectively. For 10 keV electrons, doses of 2650 and 2550 Gy (about 26 Gy per run) were obtained.

For 100 eV electrons, replacing the original cross section data with the common cross section dataset resulted in a decrease in the predicted average yields of SSB by about 6% and an increase in the yields of DSBs by about 15%, respectively. The changes are much larger than the relative statistical uncertainties of the yields, which are about 0.2% for SSBs and about 1% for DSBs. Only very rarely, a DSB cluster was scored at this low electron energy; however, the yields of DSB clusters more than tripled upon using the common cross section dataset instead of the original cross section data. None of these clusters included more than two DSBs.

For 10 keV electrons, replacing the original cross sections with the common dataset, resulted in a decrease in the predicted yields of SSB by 2.5%, while the yields of DSB increased by 0.6%, and the yields of DSB clusters increase by 7%, comparable to about twice the combined uncertainty. For both cross section datasets, the DSB clusters predominantly (in more than 98% of cases) included just two DSBs per cluster.

Key findings:

Replacing code-specific with common total interaction cross section data:

- Reduces inter-code variability in ICSD and *M*_1_ for 20 eV electrons by about 97%,
- Reduces inter-code variability in ICSD, *M*_1_, *F*_2_ and *F*_3_ variability for 50 eV electrons by about 80%,
- Reduces inter-code variability in ICSD and *M*_1_ variability for 100 eV electrons more about 67%, and in *F*_3_ by about 50%.
- Has virtually no effect on inter-code variability beyond 300 eV, indicating other factors embedded in the codes as the main reason for this variability.
- Increases predicted DSB yields for 100 eV electrons by about 15%.

## Discussion

ICSDs and related nanodosimetric quantities (e.g., mean ionization numbers or probability of two or more ionizations in a volume) were obtained for low-energy electrons with seven MCTS codes. Each code was run either with its original total interaction cross sections or a common dataset created *ad-hoc* for this study with an averaging procedure applied to initial total cross sections. The results show that total interaction cross sections, the foundation of any MCTS code, critically affect ICSDs and derived nanodosimetric quantities. Comparing ICSDs from a representative set of the most widely used MCTS codes in nanodosimetry and radiobiology modelling shows a considerable variability. This variability was quantified using two metrics: Wasserstein distance for the differences between complete ICSDs and the relative standard deviations for nanodosimetric quantities (*M*_1_, *F*_2_ and *F*_3_), as appropriate for comparing normalized discrete distributions and sets of scalar values, respectively. ICSDs differ strongly for very low-energy electrons, but the differences shrink as electron energy increases. This is shown in Table 1 and is especially visible when values are normalized by the mean *M*_1_ at each energy. These differences also depend on the target volume since the analyzed portion of the track changes with it. Thus, the present 5 keV results cannot be directly compared to 1 keV because the ICSDs were calculated in different volumes, spheres of diameter 100 nm versus 8 nm. Larger volumes include longer track portions and incorporate more low-energy interactions. The chosen volume sizes have biological relevance, 8 nm spheres approximate nucleosomes and DSB clusters, while 100 nm spheres represent chromatin loop dimensions. These choices also allow comparisons with earlier studies.

Compared with ICSDs, the nanodosimetric quantities *M*_1_, *F*_2_ and *F*_3_ show much lower variability among MCTS codes, especially at 100–300 eV. These quantities are used in nanodosimetry-based models to link track structure to biological impact at cellular level [75]. The present results suggest that the choice of MCTS code may be less critical for calculating *M*_1_, *F*_2_ and *F*_3_ than for ICSDs as a whole.

Overall, results obtained in this work highlights the strong impact of total cross section datasets on ICSD variability, especially for low-energy electrons—accounting for about 97% of the variability at 20 eV and up to 67% at 100 eV (Fig 6 and Key findings). This effect decreases as electron energy rises, with other elements of the simulation increasingly shaping track structure.

The calculation volume also affects the results: With increasing energy, the fraction of electron tracks covered by a fixed volume decreases. Larger volumes capture the tracks of fixed energy more completely. Code-specific differential cross sections mainly affect the secondary electron spectrum, and their influence on ICSDs and nanodosimetric quantities may diminish when larger track portions or even full tracks are analyzed, though this requires further verification.

For nanodosimetric quantities *(M*_1_, *F*_2_ and *F*_3_) across 20 eV - 10 keV, remaining differences are within statistical uncertainty except around 1 keV. In the small 8 nm diameter volume analyzed for 1 keV electrons, the angular dependence of the elastic cross sections strongly affects ionizations counts. Biophysically, this shows that interaction cross sections primarily drive differences in nanodosimetric quantities in volumes comparable to nucleosomes or complex DNA lesions. For electrons above ~1keV, elastic angular distributions and differential ionization cross sections can further influence these quantities by around 10–15%.

To support these conclusions, we extended our study using Geant4-DNA to modify cross sections and models. Simulations were run for options 2, 4 and 6 with the common cross section data set as previously reported and identical elastic differential cross sections (those of option 2, i.e., Champion’s model, see S1 Table in S1 File). Only the differential ionization cross sections and resulting electron angular distributions differed between the options. Results of the corresponding ICSDs are shown in S8 (right) Fig compared with the ICSDs of the original codes (a) and the codes with only the total cross sections harmonized (b). The mean values (*M*_1_) values are given in S10 Table in S1 File.

These results show that options 2 and 4 produced nearly identical ICSDs, consistent with their similar inelastic collision models based on the dielectric response function, differing only in partitioning algorithms of experimental optical data into discrete excitation and ionization shells (see S1 Table in S1 File). Option 6 still differed due to its Binary-Encounter-Bethe ionization model, though mean ICSD values (*M*_1_) remained largely unchanged.

Differences in numerical parameters such as energy cutoffs or interpolation techniques—almost identical in these 3 Geant4-DNA options—are unlikely to explain the observed variability. Cross sections are generally tabulated on dense energy grids, and interpolation choices (linear or log–log) introduce only minor discrepancies, far smaller than those associated with the physical modelling of the differential cross sections. This confirms previous findings that the choice of approximations and models needed when building a cross section database strongly affects the outcome of MCTS codes [73,74]. For instance, Emfietzoglou et al. [79] demonstrated that the way of extrapolating optical data to finite momentum transfer to calculate differential inelastic cross sections for each ionization shell was crucial in modelling the inelastic scattering of electrons with energies below 200 eV.

Given the experimental and theoretical difficulty of determining exact electron cross sections, using an average representative data set is reasonable to estimate their contribution to the uncertainty of the results. For this purpose, the specific choice of the test dataset is not critical. It is, however, important to keep in mind that the common dataset used here was derived solely for this study and was not intended to replace original cross sections, which individual codes are expected to continue using.

Nevertheless, the modulation of ICSDs from individual MCTS codes upon using the common instead of the original interaction cross sections is certainly of interest. Therefore, Wasserstein distances were calculated between the original codes and their modified versions (see S5 Table in S1 File). It can be seen, for example, that largest Wasserstein distances are obtained for the PHITS code due to differences in auto-ionization following electronic excitations in the original version (90% for the diffuse band and 100% for collective excitations).

The studied variations in MCTS outcomes are biologically relevant because low-energy electrons (< 5 keV) contribute significantly to energy deposition even by photons, about one third by ^60^Co gamma-rays and a half by 220 kVp X-rays, and DSB induction is largely due to these electrons [55]. Indeed, DNA damage simulations with PARTRAC at 100 eV and 10 keV demonstrate that different cross section datasets alter the predicted SSB and DSB yields. The two studied energies are relevant for beta decaying isotopes such as ^3^H whose mean electron energy is about 6 keV, and for Auger emitters (proposed to be called Auger-Meitner emitters) such as ^125^I that produces electrons with energies including the 50–500 eV range. The reported yields of DNA damage from the simulations are in line with previous studies assessing biological effects of diverse types of radiations [78,80,81]. At 100 eV, the common cross sections produce fewer isolated SSBs (−6%) and more DSBs (+15%) compared with the original PARTRAC cross sections. At 10 keV, differences are smaller (SSB −2.5%, DSB + 0.6%), while DSB clusters are more affected but remain rare.

These results show that differences in interaction cross sections propagate through all endpoints, from nanodosimetry and microdosimetry to DNA damage and very likely eventually to cell killing (not addressed here). If all parameters except the total interaction cross sections are kept constant, as was done here with PARTRAC, the change in the yield of DSBs can reach up to 15%. Even larger variations were reported for DSB yields predicted by alternative Geant4-DNA options for 0.3–4.5 keV electrons [82,83]. The effects may be even higher for more complex lesions such as locally multiply damage sites or DSB clusters critical in cell killing.

However, differences in DNA damage yields predicted by simulations also depend on other parameters such as the DNA geometries used, the energy threshold criteria for producing a direct or indirect break, and the very definition of a DSB or its complexity [23,84–87]. Different choices in these parameters can result in changes by a factor of more than two in the number of DSBs obtained in the simulation [24,88].

The other model parameters (relating to chemistry, biochemistry or even biology) may be used to compensate for different choices of physical parameters (e.g., interaction cross sections) so as to fit simulations to experimental results. However, this is conceptually unsatisfactory as, in general, parameters determining physical processes are known with smaller uncertainties than those governing biochemical and biological processes. Furthermore, it remains unclear to what extent differences in cross section data are compensated or amplified in such approaches. This underscores the need for further experimental and theoretical research on low-energy electron interactions in liquid water or biologically relevant media.

The present study is limited by several issues. First, the set of MCTS codes used does not cover all tools developed in the last decades for tracking low-energy electrons in liquid water. Yet it is sufficiently representative of codes actively used in radiobiological modelling and micro- and nanodosimetry. Specific reported values such as the relative standard deviation in mean ionization numbers would likely change if more codes were included. The same applies to the target volumes: The reported numerical values are valid only for the chosen volumes. Yet the qualitative and semiquantitative results would likely remain valid.

Second, the simulation setup is relatively simple, using mono-energetic electrons and spherical target volumes. The setup was intentionally kept simple to reduce potential interpretation errors by the contributors and to stay close to simulation scenarios used in previous studies (e.g., [59]). Despite the simple design, the obtained results are relevant also beyond the studied scenarios, especially for sparsely ionizing radiation where electron spurs are well separated or with radionuclides emitting low-energy electrons.

Our study focused solely on total cross sections. Since the total cross sections determine the mean free path between collisions (and resulting properties of particle tracks such as the total path travelled), it appeared reasonable to first establish their impact before investigating more complex scenarios. However, within EURADOS WG6, follow-up work has already been initiated to overcome the target volume limitation by examining ionization cluster size distributions (ICSDs) along complete electron tracks, using different cluster-associated volumes. In this ongoing effort, radial energy distributions, microdosimetric quantities, and code-specific features such as autoionization are also being investigated. In future studies, particular attention should be given to the harmonization of differential cross sections and to indirect experimental verification, especially through ion-based measurements. Despite being geometry-specific, our results indicate how the variability of total cross sections affects the variability of nanodosimetry outcomes across energies and partially also volumes. Reducing uncertainties in electron interaction cross sections would decrease nanodosimetric variability and improve DNA-scale track descriptions. In the long-term, this would enhance predictive radiobiological models, refine relative biological effectiveness (RBE) estimates for radiotherapy (including proton, ion, and FLASH modalities), and provide more reliable benchmarks for detector development and code comparisons. In radiation protection, especially in mixed or high-LET fields, this would strengthen risk assessments by reducing model-dependent variability.

As an illustrative example, simulations performed with the PARTRAC code showed a dispersion of about 15% in predicted DSB yields when different total cross section datasets were used. Although this level of variation does not directly translate into an equivalent uncertainty in predicted treatment outcomes, such microscopic-scale variability could still influence the accuracy of radiobiological models used in treatment planning. This is particularly relevant for particle therapy, where local energy deposition patterns and track-structure–based quantities underpin RBE calculations. Reducing uncertainties in low-energy electron interaction data would therefore contribute to more reliable predictions of biological effects and, ultimately, to improved confidence in biologically optimized treatment planning.

It should be emphasized, however, that the reported DNA damage simulations serve only as a sensitivity analysis, intended to illustrate the potential impact of varying total interaction cross sections on estimated biological effects. They are not meant as a validation of DSB modelling. Benchmarking of MCTS, and specifically of PARTRAC predictions against experimental data, has been discussed in detail elsewhere [18].

More generally, validation of the reported nanodosimetry results with indirect biological endpoints would be rather challenging since radiobiological experiments with monoenergetic electrons at the energies considered in this work are not available to our knowledge. Comparison with experimental ICSD data is also not easy since the only available data for low-energy electrons are those of the NCBJ (Polish National Center for Nuclear Research) group. In the papers of Bantsar et al. [57,58], target sizes equivalent to 2.3 nm in water were investigated, and the mean ionization cluster sizes were very small, comparable to those found for some codes in our work at 20 eV energy, namely 80% probability of no ionization. In [89] a radioactive source (^125^I) was used that emitted an energy spectrum, not monoenergetic electrons as used here. While it would be possible in principle to use the data produced in our work to estimate ICSDs that could be compared with this experimental work, the procedure is not trivial and would lead away from our main goal of studying to what extent the different total cross sections account for the dispersion of results with different codes.

In addition, it must be noted that the comparisons between experiment and simulations in [57,58,89] were using the MC code PTra (a variant with nitrogen gas cross sections) as a gold standard and determined the detection efficiency as a free fitting parameter to match simulations and experiments. With our agnostic or unbiased approach of taking all MCTS results as ‘equally likely to be true’, the comparison with experiments would have meant to determine for each code the corresponding detection efficiency of the Jet Counter nanodosimeter in the reported experiments, which would shift the problem of ICSD variability to a variability of detection efficiency.

Among the three reference nanodosimeters [56], the Jet Counter is the only one that has been used for experiments with electrons. The Star Track Counter features a target volume defined by an electrode arrangement that enables the detection efficiency to be determined [90]. In the Ion Counter nanodosimeter the target volume is wall-less and defined by electrical fields and the drift behavior of gas ions [91]. The instrument has been comprehensively characterized to establish its equivalent target size for liquid water when operated with different gas targets including water vapor and other gases such as DNA constituents [92]. Unfortunately, an operation with an electron gun or radionuclides emitting low-energy electrons does not appear technically feasible for either the StarTrack or the Ion Counter nanodosimeters. However, the approaches developed for the characterization of the Ion counter have already been shown to be adaptable to other types of nanodosimeters [93,94] so that corresponding developments for the Jet Counter nanodosimeter may enable independent albeit indirect experimental benchmarking of track structure simulations for liquid water in the future.

## Conclusion

The present analysis highlights the crucial role of total interaction cross sections in nanodosimetric predictions. Variability in these cross section datasets across MCTS codes strongly affects ICSDs and derived nanodosimetric quantities, especially for low-energy electrons. Using a common dataset for total interaction cross sections, created *ad-hoc* for this study by an averaging procedure, reduced the variability and indicated that total cross sections account for about 97% of the variability in ICSDs and nanodosimetric quantities at 20 eV, and up to 67% at 100 eV. Discrepancies at higher electron energies remained present, presumably mainly due to differences in assumed angular and energetic distributions of secondary electrons. This indicates that total cross sections dominate at low energies while these other factors gain importance as electron energy increases.

As a sensitivity test, DNA damage simulations using PARTRAC illustrated the biological significance of the cross-section variability. Differences in predicted SSB and DSB yields indicate that low-energy electron interactions can affect damage clustering and induction of biologically more severe lesions. Variability in DSB predictions, reaching up to 15%, underscores the need for precision in cross section modeling, particularly for sources producing abundant low-energy electrons, such as Auger (Auger-Meitner) emitters.

Because single shell interaction cross section data for low-energy electrons in condensed matter can hardly be directly validated experimentally, high-quality measurements of integrated physical quantities resulting from electron tracks – or even on biological endpoints such as DSBs – are essential. Such data would provide a more direct basis for comparing MCTS codes and improving their reliability, in the long-term enhancing predictive power of models in radiobiology, radiation protection, and medical applications.

## Supporting information

S1 File Supporting information. This file contains supplementary tables (S1–S11), supplementary figures (S1–S8), and a description of the code availability used in this study. Detailed captions for each table and figure are provided within the file.
(PDF)
