# Supplementary material for: Evaluation of the uncertainty in calculating nanodosimetric quantities due to the use of different interaction cross sections in Monte Carlo track structure codes
Source: PLoS One. 2026 Jan 9;21(1):e0340500. doi: 10.1371/journal.pone.0340500 (PMC12788675; doi:10.1371/journal.pone.0340500)
Supplement: S1 File — This file contains supplementary tables (S1–S11), supplementary figures (S1–S8), and a description of the code availability used in this study. Detailed captions for each table and figure are provided within the file. (PDF) [file pone.0340500.s001.pdf]

Supplementary Tables

Summary of the interaction models used by the MCTS codes participating in this work

Supplementary Table S1. MCTS codes used in this work and references to the physical models used to calculate interaction probabilities of electrons in liquid water within these codes. The last three columns give some detail about the code characteristics or parameters used in this work.

**Code**	**Ionization**	**Electronic excitation**	**Elastic scattering**	**E cut off (eV)**	**Lowest ionization energy (eV)**	**Interpolation method of tabulated data**
Geant4-DNA-Option 2	PWBA [1,2] (Drude functions) [a,*]	PWBA [1,2] (same as ionizations)	Partial-wave formalism [3]	7.4	10.79	log-log
Geant4-DNA-Option 4	PWBA [1,2] (Drude function algorithm) [b,*]	PWBA via the Kramers-Kronig relation [1,2] (same as ionization)	Uehara screened Rutherford model [4]	10	10	log-log
Geant4-DNA-Option 6	Relativistic BEB (CPA-100) [5]	PWBA [6,7] (same as PARTRAC)[**]	Independent Atom Method (IAM) (from the CPA100 code) [5] for T> 50 eV and experimental data on ice for T<50 eV [8]	11	10.79	log-log

PARTRAC	PWBA [6,7] (Drude function with Kramers-Kronig relation) c,*	PWBA [6,7] (same as ionization)**	NIST ELAST database values for atomic hydrogen and oxygen [9]	10	10.79	Original: 98 logarithmic equidistant values per decade; with no interpolation. Modified: log-log
MCwater	PWBA [10-12] (full Penn algorithm) d	PWBA [10-12] (same as ionization)	ELSEPA code [13] with electronic densities of [14]	10	10.79	cubic spline between linear values
PTra	BEB [15] + K shell from [16]	Tabulated values from [17]	T <200 eV: [18] T> 200 eV: [19]	10	12.61	log-log
PHITS	T < 100 keV: Tabulated values [20]; T >100 keV : Relativistic BEB [15]	T < 100 keV : Tabulated values [20] ;<br>T >100 keV : PWBA [21]	Moliere's Model [22]	1	10.9	linear-linear

PWBA: plane-wave Born approximation, BEB: Binary encounter Bethe model, CPA100: MC code of Terrisol et al. (1990)
a Optical data model based on Drude-like functions.
b Optical data model based on Drude-like functions with a different parametrization algorithm. In the present work an extended relativistic version of this algorithm has been used described in (Kyriakou et al. 2025).
c Optical data model based on Drude-like functions with a different representation of the optical data.
d Optical data model based on the Penn model.
* For K-shell ionization different models are used as described in the corresponding references.
**Different Drude parametrizations are used that can give rise to largely varying cross section values among the codes (Kyriakou et al. 2015)

References Table S-1

[1] Heller JM, Hamm RN, Birkhoff RD, Painter LR. Collective oscillation in liquid water. *J Chem Phys.* 1974;60:3483-3486. doi:10.1063/1.1681563.

[2] Kyriakou I, Incerti S, Francis Z. Improvements in Geant4 energy-loss model and the effect on low-energy electron transport in liquid water. *Med Phys.* 2015;42:3870-3876. doi:10.1118/1.4921613.

[3] Champion C, Incerti S, Aouchiche H, Oubaziz D. A free-parameter theoretical model for describing the electron elastic scattering in water in the Geant4 toolkit. *Radiat Phys Chem.* 2009;78:745-750. doi:10.1016/j.radphyschem.2009.03.079.

[4] Uehara S, Nikjoo H, Goodhead DT. Cross sections for water vapour for the Monte Carlo electron track structure code from 10 eV to the MeV region. *Phys Med Biol.* 1992;37:1841-1858. doi:10.1088/0031-9155/38/12/010.

[5] Bordage M, Bordes J, Edel S, Terrissol M, Franceries X, Bardiès M, et al. Implementation of new physics models for low energy electrons in liquid water in Geant4-DNA. *Phys Medica.* 2016;32:1833-1840. doi:10.1016/j.ejmp.2016.10.006.

[6] Dingfelder M, Hantke D, Inokuti M, Paretzke HG. Electron inelastic-scattering cross sections in liquid water. *Radiat Phys Chem.* 1998;53:1-18. doi:10.1016/S0969-806X(97)00317-4.

[7] Dingfelder M, Ritchie RH, Turner JE, Friedland W, Paretzke HG, Hamm RN. Comparisons of calculations with PARTRAC and NOREC: transport of electrons in liquid water. *Radiat Res.* 2008;169:584-594. doi:10.1667/RR1099.1.

[8] Michaud M, Sanche L. Absolute vibrational excitation cross sections for slow-electron (1-18 eV) scattering in solid H_2O. *Phys Rev A.* 1987;36(10):4684-4699. doi:10.1103/PhysRevA.36.4684.

[9] Berger MJ, Seltzer SM, Wang R, Schechter A. Elastic scattering of electrons and positrons by atoms: database ELAST. NISTIR 5166, U.S. Department of Commerce, Gaithersburg, MD; 1993.

[10] Dingfelder M. Updated model for dielectric response function of liquid water. *Appl Radiat Isot.* 2014;83:142-147. doi:10.1016/j.apradiso.2013.01.016.

[11] Hayashi H, Watanabe N, Udagawa Y, Kao CC. The complete optical spectrum of liquid water measured by inelastic x-ray scattering. *Proc Natl Acad Sci USA.* 2000;97:6264-6266. doi:10.1073/pnas.110572097.

[12] Penn DR. Electron mean-free-path calculations using a model dielectric function. *Phys Rev B.* 1987;35(2):482. doi:10.1103/PhysRevB.35.482.

[13] Salvat F, Jablonski A, Powell CJ. ELSEPA — Dirac partial-wave calculation of elastic scattering of electrons and positrons by atoms, positive ions and molecules. *Comput Phys Commun.* 2005;165:157-190. doi:10.1016/j.cpc.2020.107704.

[14] Neuefeind J, Benmore CJ, Tomberli B, Egelstaff PA. Experimental determination of the electron density of liquid H_2O and D_2O. *J Phys Condens Matter.* 2002;14(23):L429. doi:10.1088/0953-8984/14/23/104.

[15] Kim YK, Santos JP, Parente F. Extension of the binary-encounter-dipole model to relativistic incident electrons. *Phys Rev A.* 2000;62:052710. doi:10.1103/PhysRevA.62.052710.

[16] Kaplan IG, Miterev AM, Sukhonosov VYa. Simulation of the primary stage of liquid water radiolysis. *Radiat Phys Chem.* 1990;36(3):493-498. doi:10.1016/1359-0197(90)90039-K.

[17] Bigildeev EA, Michalik V. Charged particle tracks in water of different phases: Monte Carlo simulation of electron tracks. *Radiat Phys Chem.* 1996;47:197-207. doi:10.1016/0969-806X(95)00002-F .

[18] Brenner DJ, Zaider A. A computationally convenient parameterisation of experimental angular distributions of low energy electrons elastically scattered off water vapour. *Phys Med Biol.* 1984;29:443-447. doi:10.1088/0031-9155/29/4/015.

[19] Grosswendt B, Waibel E. Transport of low energy electrons in nitrogen and air. *Nucl Instrum Meth.* 1978;155:145-156. doi:10.1016/0029-554X(78)90198-2.

[20] (Kai T, Toigawa T, Matsuya Y, et al. Significant role of secondary electrons in the formation of a multi-body chemical species spur produced by water radiolysis. *Sci Rep.* 2024;14:24722. doi:10.1038/s41598-024-76481-z.

[21] Paretzke HG. Simulation von Elektronenspuren in Energiebereich 0.01–10 keV in Wasserdampf. GSF-Bericht; 1988. p.1-87.

[22] Moliere G. Theorie der Streuung schneller geladener Teilchen II: Mehrfach- und Vielfachstreuung. *Z Naturforsch A.* 1948;3a:78-97. doi:10.1515/zna-1948-0203.

Participants' data with original codes

Supplementary Table S2. Original participants data on ICSDs (limited to 28 ionizations).

Energy (eV)	ICS	G4DNA- 2	G4DNA- 4	G4DNA- 6	PARTRAC	MCwater	PTra	PHITS
20	0	2.32E-01	5.63E-01	1.67E-01	7.92E-01	7.03E-01	3.78E-01	5.45E-01
20	1	7.68E-01	4.37E-01	8.33E-01	2.08E-01	2.97E-01	6.22E-01	4.55E-01
50	0	9.72E-03	1.61E-02	2.01E-03	1.15E-01	6.09E-02	1.22E-02	1.83E-03
50	1	1.01E-01	2.98E-01	6.48E-02	5.71E-01	4.99E-01	2.47E-01	1.78E-01
50	2	5.66E-01	5.85E-01	4.52E-01	3.02E-01	4.00E-01	6.27E-01	7.36E-01
50	3	3.20E-01	9.96E-02	4.66E-01	1.27E-02	3.98E-02	1.14E-01	8.43E-02
50	4	3.32E-03	7.20E-04	1.60E-02	0.00E+00	6.00E-05	0.00E+00	0.00E+00
100	0	1.13E-02	1.34E-02	2.85E-03	1.54E-02	9.60E-03	9.13E-03	6.71E-04
100	1	2.66E-02	3.60E-02	8.57E-03	6.95E-02	3.55E-02	2.76E-02	3.26E-03
100	2	5.09E-02	8.10E-02	2.00E-02	2.64E-01	1.79E-01	7.33E-02	1.53E-02
100	3	1.15E-01	2.59E-01	6.43E-02	4.21E-01	4.07E-01	2.49E-01	1.49E-01
100	4	2.93E-01	4.05E-01	2.32E-01	2.04E-01	2.96E-01	4.19E-01	5.54E-01
100	5	3.69E-01	1.84E-01	4.04E-01	2.54E-02	6.88E-02	2.05E-01	2.70E-01
100	6	1.28E-01	2.23E-02	2.39E-01	6.80E-04	4.17E-03	1.65E-02	7.56E-03
100	7	6.65E-03	6.20E-04	2.97E-02	1.0E-05	1.00E-05	0.00E+00	4.94E-06
300	0	7.70E-02	8.73E-02	3.77E-02	6.66E-02	7.66E-02	6.76E-02	1.82E-02
300	1	1.09E-01	1.32E-01	7.62E-02	1.21E-01	1.05E-01	1.17E-01	5.73E-02
300	2	1.20E-01	1.39E-01	9.99E-02	1.39E-01	1.12E-01	1.30E-01	9.85E-02
300	3	1.13E-01	1.27E-01	1.02E-01	1.32E-01	1.04E-01	1.21E-01	1.22E-01
300	4	9.99E-02	1.05E-01	9.67E-02	1.13E-01	9.36E-02	1.04E-01	1.23E-01
300	5	8.38E-02	8.46E-02	8.49E-02	9.48E-02	8.06E-02	8.53E-02	1.10E-01
300	6	6.98E-02	6.65E-02	7.39E-02	7.63E-02	6.94E-02	6.94E-02	9.08E-02
300	7	5.76E-02	5.32E-02	6.21E-02	6.63E-02	6.35E-02	5.45E-02	7.17E-02
300	8	4.77E-02	4.26E-02	5.27E-02	5.95E-02	6.40E-02	4.71E-02	5.59E-02
300	9	4.01E-02	3.62E-02	4.46E-02	5.22E-02	6.55E-02	4.24E-02	4.51E-02
300	10	3.50E-02	3.36E-02	3.91E-02	4.17E-02	6.30E-02	4.12E-02	3.94E-02
300	11	3.15E-02	3.23E-02	3.63E-02	2.49E-02	5.30E-02	4.16E-02	4.13E-02
300	12	3.01E-02	2.83E-02	3.42E-02	9.81E-03	3.19E-02	3.68E-02	4.92E-02
300	13	2.88E-02	1.94E-02	3.77E-02	2.59E-03	1.36E-02	2.55E-02	4.71E-02
300	14	2.48E-02	9.64E-03	3.88E-02	3.90E-04	3.68E-03	1.21E-02	2.44E-02
300	15	1.82E-02	2.96E-03	3.57E-02	8.00E-05	6.50E-04	3.38E-03	5.05E-03
300	16	9.61E-03	6.80E-04	2.70E-02	0.00E+00	7.00E-05	6.50E-04	3.43E-04
300	17	3.12E-03	4.00E-05	1.42E-02	0.00E+00	0.00E+00	4.00E-05	1.71E-05
300	18	5.40E-04	1.00E-05	5.10E-03	0.00E+00	0.00E+00	0.00E+00	0.00E+00
300	19	1.00E-04	0.00E+00	1.26E-03	0.00E+00	0.00E+00	0.00E+00	0.00E+00
300	20	0.00E+00	0.00E+00	1.70E-04	0.00E+00	0.00E+00	0.00E+00	0.00E+00
600	0	2.12E-01	2.20E-01	1.35E-01	2.02E-01	2.13E-01	1.94E-01	8.06E-02
600	1	1.98E-01	2.25E-01	1.81E-01	2.33E-01	1.95E-01	2.23E-01	1.70E-01
600	2	1.66E-01	1.76E-01	1.70E-01	1.87E-01	1.58E-01	1.78E-01	1.95E-01
600	3	1.22E-01	1.23E-01	1.34E-01	1.32E-01	1.19E-01	1.24E-01	1.65E-01

Energy (eV)	ICS	G4DNA- 2	G4DNA- 4	G4DNA- 6	PARTRAC	MCwater	PTra	PHITS
600	4	8.64E-02	8.28E-02	1.01E-01	8.27E-02	8.61E-02	8.56E-02	1.17E-01
600	5	6.11E-02	5.45E-02	7.38E-02	5.53E-02	6.27E-02	5.91E-02	7.92E-02
600	6	4.23E-02	3.68E-02	5.33E-02	3.49E-02	4.48E-02	3.97E-02	5.11E-02
600	7	3.03E-02	2.51E-02	3.76E-02	2.28E-02	3.14E-02	2.68E-02	3.35E-02
600	8	2.28E-02	1.76E-02	2.75E-02	1.56E-02	2.37E-02	1.93E-02	2.40E-02
600	9	1.54E-02	1.19E-02	2.09E-02	1.07E-02	1.68E-02	1.35E-02	1.73E-02
600	10	1.08E-02	8.55E-03	1.57E-02	7.55E-03	1.17E-02	1.02E-02	1.29E-02
600	11	8.23E-03	5.78E-03	1.13E-02	4.77E-03	9.14E-03	6.91E-03	1.07E-02
600	12	6.26E-03	3.85E-03	8.66E-03	3.47E-03	6.35E-03	5.25E-03	8.70E-03
600	13	4.51E-03	2.85E-03	6.79E-03	2.21E-03	5.10E-03	3.90E-03	7.41E-03
600	14	3.50E-03	2.05E-03	5.18E-03	1.67E-03	4.25E-03	2.99E-03	5.76E-03
600	15	2.33E-03	1.63E-03	4.17E-03	1.47E-03	3.06E-03	2.10E-03	4.91E-03
600	16	1.92E-03	9.30E-04	3.19E-03	1.10E-03	2.49E-03	1.53E-03	3.78E-03
600	17	1.69E-03	7.10E-04	2.54E-03	6.5E-04	1.83E-03	1.27E-03	3.28E-03
600	18	1.22E-03	5.20E-04	1.97E-03	5.40E-04	1.51E-03	9.20E-04	2.48E-03
600	19	7.90E-04	4.20E-04	1.66E-03	2.80E-04	1.22E-03	6.20E-04	2.05E-03
600	20	7.50E-04	2.60E-04	1.28E-03	3.00E-04	8.10E-04	4.10E-04	1.74E-03
600	21	5.40E-04	1.10E-04	1.07E-03	1.20E-04	5.40E-04	5.60E-04	1.24E-03
600	22	3.40E-04	1.50E-04	8.40E-04	8.00E-05	3.90E-04	2.40E-04	9.46E-04
600	23	2.70E-04	1.50E-04	6.80E-04	4.00E-05	1.80E-04	2.20E-04	8.94E-04
600	24	2.90E-04	9.00E-05	5.70E-04	0.00E+00	1.00E-04	1.80E-04	7.06E-04
600	25	1.50E-04	5.00E-05	5.70E-04	0.00E+00	4.00E-05	1.40E-04	5.63E-04
600	26	1.20E-04	4.00E-05	3.80E-04	0.00E+00	2.00E-05	5.00E-05	4.47E-04
600	27	1.40E-04	2.00E-05	2.60E-04	0.00E+00	0.00E+00	1.00E-05	2.85E-04
600	28	4.00E-05	0.00E+00	2.60E-04	0.00E+00	0.00E+00	0.00E+00	1.30E-04
1 000	0	3.54E-01	3.53E-01	2.58E-01	3.47E-01	3.45E-01	3.31E-01	1.88E-01
1 000	1	2.29E-01	2.54E-01	2.40E-01	2.72E-01	2.27E-01	2.60E-01	2.74E-01
1 000	2	1.52E-01	1.57E-01	1.74E-01	1.65E-01	1.52E-01	1.63E-01	2.21E-01
1 000	3	9.51E-02	9.27E-02	1.12E-01	9.20E-02	9.82E-02	9.48E-02	1.34E-01
1 000	4	5.93E-02	5.47E-02	7.23E-02	5.09E-02	6.34E-02	5.55E-02	7.53E-02
1 000	5	3.79E-02	3.29E-02	4.62E-02	2.85E-02	3.92E-02	3.41E-02	3.90E-02
1 000	6	2.43E-02	2.02E-02	3.05E-02	1.71E-02	2.54E-02	2.16E-02	2.22E-02
1 000	7	1.64E-02	1.26E-02	2.08E-02	1.08E-02	1.66E-02	1.32E-02	1.37E-02
1 000	8	1.04E-02	8.40E-03	1.43E-02	6.50E-03	1.13E-02	9.05E-03	9.11E-03
1 000	9	6.92E-03	5.19E-03	9.55E-03	3.94E-03	6.91E-03	6.14E-03	6.05E-03
1 000	10	4.60E-03	3.77E-03	6.72E-03	2.38E-03	4.77E-03	3.86E-03	4.54E-03
1 000	11	3.24E-03	1.89E-03	4.88E-03	1.69E-03	3.27E-03	2.90E-03	3.34E-03
1 000	12	2.14E-03	1.34E-03	3.25E-03	9.60E-04	1.94E-03	1.81E-03	2.16E-03
1 000	13	1.45E-03	9.10E-04	2.38E-03	6.40E-04	1.31E-03	1.11E-03	1.71E-03
1 000	14	1.00E-03	6.80E-04	1.63E-03	5.30E-04	9.70E-04	7.90E-04	1.25E-03
1 000	15	6.30E-04	4.90E-04	1.35E-03	2.10E-04	7.80E-04	6.20E-04	1.17E-03
1 000	16	4.20E-04	1.70E-04	7.60E-04	1.30E-04	4.20E-04	4.20E-04	6.72E-04
1 000	17	3.20E-04	1.40E-04	6.20E-04	1.20E-04	3.60E-04	2.80E-04	6.18E-04
1 000	18	1.50E-04	1.30E-04	5.30E-04	1.30E-04	2.00E-04	2.30E-04	4.37E-04

Energy (eV)	ICS	G4DNA- 2	G4DNA- 4	G4DNA- 6	PARTRAC	MCwater	PTra	PHITS
1 000	19	1.10E-04	7.00E-05	3.40E-04	8.00E-05	1.80E-04	1.40E-04	4.69E-04
1 000	20	1.40E-04	8.00E-05	2.50E-04	3.00E-05	1.40E-04	7.00E-05	1.92E-04
1 000	21	4.00E-05	3.00E-05	2.30E-04	5.00E-05	5.00E-05	6.00E-05	3.09E-04
1 000	22	1.00E-04	2.00E-05	9.00E-05	1.00E-05	2.00E-05	3.00E-05	6.40E-05
1 000	23	0.00E+00	3.00E-05	3.00E-05	1.00E-05	1.00E-05	4.00E-05	1.81E-04
1 000	24	3.00E-05	2.00E-05	8.00E-05	0.00E+00	3.00E-05	2.00E-05	7.46E-05
1 000	25	3.00E-05	1.00E-05	5.00E-05	0.00E+00	2.00E-05	0.00E+00	7.46E-05
1 000	26	3.00E-05	0.00E+00	4.00E-05	1.00E-05	2.00E-05	1.00E-05	5.33E-05
1 000	27	0.00E+00	0.00E+00	3.00E-05	0.00E+00	0.00E+00	0.00E+00	4.26E-05
1 000	28	0.00E+00	1.00E-05	1.00E-05	0.00E+00	2.00E-05	0.00E+00	6.40E-05
5 000	0	3.34E-02	2.95E-02	1.18E-02	2.75E-02	3.65E-02	2.55E-02	4.52E-03
5 000	1	6.33E-02	6.89E-02	3.41E-02	7.07E-02	7.29E-02	6.63E-02	1.87E-02
5 000	2	9.06E-02	9.90E-02	6.07E-02	1.04E-01	9.83E-02	9.84E-02	4.79E-02
5 000	3	1.01E-01	1.13E-01	8.13E-02	1.16E-01	1.05E-01	1.12E-01	8.01E-02
5 000	4	1.00E-01	1.11E-01	9.41E-02	1.09E-01	1.04E-01	1.11E-01	1.03E-01
5 000	5	9.28E-02	1.02E-01	9.51E-02	9.89E-02	9.70E-02	1.02E-01	1.15E-01
5 000	6	8.31E-02	8.74E-02	9.23E-02	8.28E-02	8.11E-02	8.58E-02	1.13E-01
5 000	7	7.00E-02	7.23E-02	8.34E-02	6.57E-02	6.89E-02	7.10E-02	1.02E-01
5 000	8	6.06E-02	5.69E-02	7.34E-02	5.11E-02	5.52E-02	5.77E-02	8.20E-02
5 000	9	5.02E-02	4.58E-02	6.10E-02	4.16E-02	4.44E-02	4.63E-02	6.50E-02
5 000	10	4.07E-02	3.77E-02	5.16E-02	3.19E-02	3.66E-02	3.53E-02	4.59E-02
5 000	11	3.43E-02	3.02E-02	4.33E-02	2.54E-02	2.87E-02	2.90E-02	3.57E-02
5 000	12	2.78E-02	2.60E-02	3.63E-02	2.06E-02	2.32E-02	2.37E-02	2.78E-02
5 000	13	2.36E-02	2.01E-02	2.90E-02	1.60E-02	1.85E-02	1.89E-02	2.02E-02
5 000	14	1.97E-02	1.61E-02	2.42E-02	1.39E-02	1.55E-02	1.50E-02	1.51E-02
5 000	15	1.59E-02	1.33E-02	1.99E-02	1.12E-02	1.28E-02	1.29E-02	1.09E-02
5 000	16	1.40E-02	1.08E-02	1.78E-02	9.68E-03	1.05E-02	1.05E-02	9.37E-03
5 000	17	1.27E-02	9.24E-03	1.49E-02	8.25E-03	8.83E-03	9.09E-03	6.42E-03
5 000	18	1.01E-02	8.16E-03	1.24E-02	7.28E-03	8.09E-03	7.66E-03	5.30E-03
5 000	19	8.90E-03	6.83E-03	1.05E-02	6.59E-03	6.70E-03	6.33E-03	5.22E-03
5 000	20	7.81E-03	5.70E-03	8.23E-03	5.67E-03	6.05E-03	5.71E-03	4.81E-03
5 000	21	6.77E-03	4.99E-03	8.21E-03	5.24E-03	5.06E-03	5.16E-03	3.47E-03
5 000	22	5.63E-03	4.65E-03	6.62E-03	5.26E-03	4.28E-03	3.89E-03	3.15E-03
5 000	23	5.29E-03	3.91E-03	5.70E-03	4.66E-03	3.77E-03	3.99E-03	2.92E-03
5 000	24	4.65E-03	3.87E-03	5.41E-03	4.26E-03	3.27E-03	3.33E-03	2.87E-03
5 000	25	4.04E-03	3.26E-03	4.48E-03	3.85E-03	3.32E-03	3.18E-03	2.06E-03
5 000	26	3.70E-03	2.65E-03	3.98E-03	3.98E-03	2.93E-03	2.60E-03	2.32E-03
5 000	27	3.45E-03	2.69E-03	3.78E-03	3.63E-03	2.64E-03	2.28E-03	2.38E-03
5 000	28	3.07E-03	2.31E-03	3.51E-03	3.06E-03	2.47E-03	2.07E-03	2.09E-03
10 000	0	1.50E-01	1.19E-01	8.35E-02	1.41E-01	1.53E-01	1.36E-01	4.84E-02
10 000	1	1.62E-01	1.71E-01	1.42E-01	1.93E-01	1.75E-01	1.89E-01	1.27E-01
10 000	2	1.55E-01	1.67E-01	1.55E-01	1.76E-01	1.61E-01	1.74E-01	1.74E-01
10 000	3	1.27E-01	1.36E-01	1.39E-01	1.33E-01	1.28E-01	1.35E-01	1.77E-01
10 000	4	9.57E-02	1.04E-01	1.12E-01	9.62E-02	9.58E-02	9.68E-02	1.40E-01

Energy (eV)	ICS	G4DNA- 2	G4DNA- 4	G4DNA- 6	PARTRAC	MCwater	PTra	PHITS
10 000	5	7.11E-02	7.50E-02	8.65E-02	6.39E-02	6.89E-02	6.84E-02	9.89E-02
10 000	6	5.23E-02	5.31E-02	6.48E-02	4.28E-02	4.81E-02	4.69E-02	6.46E-02
10 000	7	3.94E-02	3.86E-02	4.82E-02	2.94E-02	3.57E-02	3.33E-02	4.04E-02
10 000	8	2.91E-02	2.89E-02	3.53E-02	2.02E-02	2.52E-02	2.46E-02	2.61E-02
10 000	9	2.27E-02	2.13E-02	2.69E-02	1.48E-02	1.92E-02	1.76E-02	1.65E-02
10 000	10	1.63E-02	1.60E-02	2.06E-02	1.08E-02	1.34E-02	1.28E-02	1.20E-02
10 000	11	1.39E-02	1.20E-02	1.55E-02	8.53E-03	1.06E-02	1.01E-02	8.40E-03
10 000	12	1.09E-02	9.46E-03	1.25E-02	7.34E-03	8.36E-03	8.06E-03	6.11E-03
10 000	13	8.47E-03	7.62E-03	9.53E-03	5.43E-03	6.43E-03	6.01E-03	4.12E-03
10 000	14	6.61E-03	6.44E-03	7.63E-03	5.13E-03	5.28E-03	4.71E-03	4.00E-03
10 000	15	6.24E-03	4.98E-03	6.26E-03	3.99E-03	4.88E-03	4.18E-03	3.22E-03
10 000	16	5.07E-03	4.01E-03	5.11E-03	3.51E-03	3.85E-03	3.66E-03	2.85E-03
10 000	17	4.32E-03	3.61E-03	4.40E-03	2.82E-03	3.24E-03	2.89E-03	2.06E-03
10 000	18	3.64E-03	3.30E-03	4.14E-03	2.92E-03	2.70E-03	2.71E-03	1.78E-03
10 000	19	3.17E-03	3.07E-03	2.93E-03	2.68E-03	2.63E-03	2.42E-03	1.46E-03
10 000	20	2.55E-03	2.38E-03	3.06E-03	2.69E-03	2.22E-03	2.02E-03	1.18E-03
10 000	21	2.57E-03	2.26E-03	2.65E-03	2.49E-03	2.18E-03	1.72E-03	1.02E-03
10 000	22	1.91E-03	1.63E-03	2.15E-03	2.42E-03	1.69E-03	1.43E-03	1.13E-03
10 000	23	1.84E-03	1.62E-03	1.82E-03	2.18E-03	1.59E-03	1.50E-03	1.16E-03
10 000	24	1.91E-03	1.60E-03	1.82E-03	2.13E-03	1.49E-03	1.22E-03	9.95E-04
10 000	25	1.65E-03	1.54E-03	1.62E-03	1.90E-03	1.25E-03	1.09E-03	1.48E-03
10 000	26	1.31E-03	1.29E-03	1.37E-03	1.78E-03	1.32E-03	1.01E-03	1.13E-03
10 000	27	1.35E-03	1.19E-03	1.07E-03	1.48E-03	1.46E-03	9.60E-04	1.25E-03
10 000	28	1.15E-03	1.18E-03	1.15E-03	1.48E-03	1.16E-03	8.10E-04	1.74E-03

Wasserstein distances between ICSDs with original and modified codes

Supplementary Table S3. Wasserstein distances calculated between each original ICSD and the mean ICSD presented in black color in Figure 2 and Figure 3.

Energy (eV)	G4DNA-2	G4DNA-4	G4DNA-6	PARTRAC	MCwater	PTra	PHITS	Mean value
**20**	0.251 ± 0.002	0.080 ± 0.002	0.316 ± 0.002	0.309 ± 0.002	0.220 ± 0.002	0.105 ± 0.002	0.062 ± 0.002	0.19 ± 0.04
**50**	0.380 ± 0.003	0.084 ± 0.003	0.602 ± 0.003	0.616 ± 0.003	0.407 ± 0.003	0.125 ± 0.003	0.244 ± 0.002	0.35 ± 0.08
**100**	0.512 ± 0.004	0.164 ± 0.004	1.033 ± 0.004	0.988 ± 0.003	0.628 ± 0.003	0.157 ± 0.004	0.433 ± 0.003	0.56 ± 0.13
**300**	0.228 ± 0.007	0.784 ± 0.006	1.330 ± 0.007	0.889 ± 0.006	0.329 ± 0.007	0.303 ± 0.007	0.784 ± 0.007	0.66 ± 0.15
**600**	0.100 ± 0.006	0.475 ± 0.005	0.567 ± 0.006	0.502 ± 0.005	0.147 ± 0.006	0.238 ± 0.005	0.733 ± 0.006	0.39 ± 0.09
**1000**	0.098 ± 0.005	0.217 ± 0.005	0.375 ± 0.005	0.321 ± 0.004	0.103 ± 0.005	0.132 ± 0.005	0.332 ± 0.005	0.23 ± 0.04
**5000**	0.343 ± 0.008	0.861 ± 0.008	1.027 ± 0.008	0.654 ± 0.008	0.422 ± 0.008	0.420 ± 0.008	1.148 ± 0.008	0.70 ± 0.12
**10000**	0.263 ± 0.007	0.199 ± 0.007	0.595 ± 0.007	0.469 ± 0.007	0.238 ± 0.007	0.374 ± 0.007	0.663 ± 0.007	0.40 ± 0.07

Supplementary Table S4. Wasserstein distances calculated between each ICSD obtained with modified MCTS codes and the mean ICSD presented in black color in Figure 4 and Figure 5.

Energy (eV)	G4DNA-2	G4DNA-4	G4DNA-6	PARTRAC	MCwater	PTra	PHITS	Mean value
**20**	0.013 ± 0.002	0.002 ± 0.002	0.002 ± 0.002	0.010 ± 0.002	0.007 ± 0.002	0.002 ± 0.002	0.012 ± 0.002	0.007 ± 0.002
**50**	0.043 ± 0.003	0.023 ± 0.003	0.096 ± 0.003	0.039 ± 0.003	0.153 ± 0.003	0.089 ± 0.003	0.132 ± 0.003	0.082 ± 0.003
**100**	0.091 ± 0.004	0.036 ± 0.004	0.166 ± 0.004	0.119 ± 0.003	0.340 ± 0.004	0.242 ± 0.004	0.286 ± 0.004	0.183 ± 0.004
**300**	0.498 ± 0.007	0.157 ± 0.006	0.328 ± 0.007	0.134 ± 0.007	0.306 ± 0.007	0.659 ± 0.006	0.164 ± 0.007	0.321 ± 0.007
**600**	0.260 ± 0.006	0.225 ± 0.006	0.389 ± 0.005	0.261 ± 0.006	0.317 ± 0.006	0.617 ± 0.005	0.526 ± 0.006	0.371 ± 0.006
**1000**	0.247 ± 0.005	0.235 ± 0.005	0.235 ± 0.005	0.257 ± 0.005	0.287 ± 0.005	0.484 ± 0.005	0.107 ± 0.005	0.265 ± 0.005
**5000**	0.253 ± 0.008	0.299 ± 0.008	0.771 ± 0.008	0.442 ± 0.008	0.603 ± 0.008	1.344 ± 0.008	0.631 ± 0.008	0.620 ± 0.008
**10000**	0.274 ± 0.007	0.302 ± 0.007	0.330 ± 0.007	0.311 ± 0.007	0.475 ± 0.007	0.961 ± 0.007	0.156 ± 0.007	0.401 ± 0.007

Supplementary Table S5. Wasserstein distances calculated between each ICSD obtained with the original MCTS code and its modified version using common cross sections used in this work.

Energy (eV)	G4DNA-2	G4DNA-4	G4DNA-6	PARTRAC	MCwater	PTra	PHITS
**20**	0.170 ± 0.002	0.175 ± 0.002	0.225 ± 0.002	0.413 ± 0.002	0.306 ± 0.002	0.014 ± 0.002	0.470 ± 0.002
**50**	0.382 ± 0.003	0.078 ± 0.003	0.493 ± 0.003	0.645 ± 0.003	0.337 ± 0.003	0.083 ± 0.003	1.202 ± 0.003
**100**	0.579 ± 0.004	0.081 ± 0.003	0.948 ± 0.004	1.019 ± 0.003	0.419 ± 0.004	0.250 ± 0.003	2.287 ± 0.003
**300**	1.331 ± 0.007	1.626 ± 0.006	0.610 ± 0.007	1.751 ± 0.006	1.175 ± 0.006	0.495 ± 0.006	3.609 ± 0.006
**600**	0.473 ± 0.006	0.693 ± 0.005	0.798 ± 0.006	0.406 ± 0.005	0.389 ± 0.006	0.268 ± 0.005	2.224 ± 0.005
**1000**	0.478 ± 0.005	0.622 ± 0.005	0.385 ± 0.005	0.259 ± 0.005	0.416 ± 0.005	0.185 ± 0.004	1.296 ± 0.004
**5000**	0.917 ± 0.008	1.520 ± 0.008	1.462 ± 0.008	0.755 ± 0.008	1.192 ± 0.008	0.540 ± 0.008	4.881 ± 0.007
**10000**	0.772 ± 0.007	0.849 ± 0.007	0.627 ± 0.007	0.515 ± 0.007	0.935 ± 0.007	0.230 ± 0.006	2.795 ± 0.006

Nanodosimetric quantities results: F_3 values (cumulative probability of obtaining 3 or more ionizations)

Supplementary Table S6. F_3 values obtained with original MCTS codes, and the corresponding mean (MV), standard deviation (SD) and relative standard deviation (RSD) of this set of codes.

Energy (eV)	G4DNA-2	G4DNA-4	G4DNA-6	PARTRAC	MCwater	PTra	PHITS	MV	SD	RSD
**50**	0.323 ± 0.001	0.100 ± 0.001	0.482 ± 0.002	0.013 ± 0.000	0.040 ± 0.001	0.114 ± 0.001	0.084 ± 0.001	0.17 ± 0.07	0.1719 ± 0.0010	1.041 ± 0.004
**100**	0.912 ± 0.001	0.871 ± 0.001	0.969 ± 0.001	0.651 ± 0.002	0.776 ± 0.001	0.890 ± 0.001	0.981 ± 0.000	0.86 ± 0.05	0.1159 ± 0.0010	0.1342 ± 0.0004
**300**	0.694 ± 0.001	0.642 ± 0.002	0.786 ± 0.001	0.674 ± 0.001	0.707 ± 0.001	0.685 ± 0.001	0.825 ± 0.001	0.72 ± 0.03	0.0657 ± 0.0010	0.0917 ± 0.0004
**600**	0.424 ± 0.002	0.380 ± 0.002	0.515 ± 0.002	0.378 ± 0.002	0.433 ± 0.002	0.406 ± 0.002	0.556 ± 0.002	0.44 ± 0.03	0.0679 ± 0.0010	0.1538 ± 0.0006
**1000**	0.265 ± 0.001	0.236 ± 0.001	0.329 ± 0.001	0.217 ± 0.001	0.276 ± 0.001	0.247 ± 0.001	0.317 ± 0.001	0.269 ± 0.017	0.0414 ± 0.0010	0.1538 ± 0.0008
**5000**	0.813 ± 0.001	0.803 ± 0.001	0.893 ± 0.001	0.798 ± 0.001	0.792 ± 0.001	0.810 ± 0.001	0.929 ± 0.001	0.83 ± 0.02	0.0542 ± 0.0010	0.0650 ± 0.0003
**10000**	0.533 ± 0.002	0.543 ± 0.002	0.620 ± 0.002	0.490 ± 0.002	0.511 ± 0.002	0.501 ± 0.002	0.651 ± 0.002	0.55 ± 0.03	0.0617 ± 0.0010	0.1122 ± 0.0005

Supplementary Table S7. F_3 values obtained with modified MCTS codes using the common cross sections, and the corresponding mean (MV), standard deviation (SD) and relative standard deviation (RSD) of this set of codes.

Energy (eV)	G4DNA-2	G4DNA-4	G4DNA-6	PARTRAC	MCwater	PTra	PHITS	MV	SD	RSD
**50**	0.138 ± 0.001	0.157 ± 0.001	0.215 ± 0.001	0.156 ± 0.001	0.170 ± 0.001	0.105 ± 0.001	0.220 ± 0.001	0.166 ± 0.017	0.0410 ± 0.0002	0.2472 ± 0.0014
**100**	0.890 ± 0.001	0.862 ± 0.001	0.879 ± 0.001	0.903 ± 0.001	0.744 ± 0.001	0.831 ± 0.001	0.910 ± 0.001	0.86 ± 0.02	0.0576 ± 0.0003	0.0669 ± 0.0003
**300**	0.756 ± 0.001	0.729 ± 0.001	0.685 ± 0.001	0.697 ± 0.001	0.732 ± 0.001	0.652 ± 0.002	0.709 ± 0.001	0.708 ± 0.014	0.0343 ± 0.0002	0.0485 ± 0.0003
**600**	0.502 ± 0.002	0.495 ± 0.002	0.434 ± 0.002	0.419 ± 0.002	0.493 ± 0.002	0.371 ± 0.002	0.492 ± 0.002	0.46 ± 0.02	0.0504 ± 0.0002	0.1100 ± 0.0005
**1000**	0.333 ± 0.001	0.343 ± 0.002	0.282 ± 0.001	0.260 ± 0.001	0.348 ± 0.002	0.217 ± 0.001	0.293 ± 0.001	0.297 ± 0.020	0.0484 ± 0.0002	0.1631 ± 0.0008
**5000**	0.859 ± 0.001	0.859 ± 0.001	0.832 ± 0.001	0.821 ± 0.001	0.852 ± 0.001	0.781 ± 0.001	0.872 ± 0.001	0.839 ± 0.013	0.0310 ± 0.0002	0.0369 ± 0.0002
**10000**	0.608 ± 0.002	0.609 ± 0.002	0.569 ± 0.002	0.540 ± 0.002	0.618 ± 0.002	0.479 ± 0.002	0.587 ± 0.002	0.57 ± 0.02	0.0493 ± 0.0002	0.0861 .0004

Summary of mean nanodosimetric results obtained in this work

Supplementary Table S8. Summary table on inter-code variability values with original cross sections and with the common set: $W_1/MV(M_1)$ and RSDs for M_1, F_2, F_3.

Energy (eV)	***$W_1/MV(M_1)$***		***RSD (M_1)***		***RSD (F_2)***		***RSD (F_3)***	
	original cross sections	common-set	original cross sections	common-set	original cross sections	common-set	original cross sections	common-set
**20**	0.37 ± 0.11	0.011 ± 0.005	0.4521 ± 0.0010	0.0145 ± 0.0009	-	-	-	-
**50**	0.19 ± 0.05	0.045 ± 0.011	0.2301 ± 0.0004	0.0518 ± 0.0005	0.3363 ± 0.0008	0.0681 ± 0.0007	1.041 ± 0.003	0.247 ± 0.002
**100**	0.15 ± 0.04	0.049 ± 0.012	0.1804 ± 0.0003	0.0599 ± 0.0004	0.0277 ± 0.0002	0.0283 ± 0.0003	0.1342 ± 0.0005	0.0669 ± 0.0005
**300**	0.12 ± 0.03	0.061 ± 0.015	0.1493 ± 0.0008	0.0661 ± 0.0008	0.0602 ± 0.0004	0.0317 ± 0.0005	0.0917 ± 0.0007	0.0485 ± 0.0007
**600**	0.13 ± 0.03	0.12 ± 0.02	0.1633 ± 0.0012	0.1271 ± 0.0011	0.1167 ± 0.0008	0.0778 ± 0.0009	0.1538 ± 0.0013	0.1100 ± 0.0012
**1000**	0.12 ± 0.03	0.13 ± 0.02	0.1380 ± 0.0013	0.1368 ± 0.0012	0.1342 ± 0.0013	0.1231 ± 0.0012	0.1538 ± 0.0018	0.1631 ± 0.0017
**5000**	0.100 ± 0.019	0.09 ± 0.02	0.0883 ± 0.0008	0.0800 ± 0.0008	0.0353 ± 0.0003	0.0205 ± 0.0003	0.0650 ± 0.0004	0.0369 ± 0.0005
**10000**	0.102 ± 0.019	0.10 ± 0.03	0.0843 ± 0.0012	0.0936 ± 0.0011	0.0849 ± 0.0007	0.0620 ± 0.0007	0.1122 ± 0.0010	0.0861 ± 0.0010

Supplementary Table S9. Summary table of the mean values of M1, F2 and F3 obtained with original versus modified TSMC code in this work.

Energy (eV)	***M_1***		***F_2***		***F_3***	
	original cross-sections	common-set	original cross-sections	common-set	original cross-sections	common-set
**20**	0.52 ± 0.10	0.611 ± 0.004	-	-	-	-
**50**	1.83 ± 0.17	1.84 ± 0.04	0.69 ± 0.09	0.703 ± 0.020	0.17 ± 0.07	0.166 ± 0.017
**100**	3.8 ± 0.3	3.72 ± 0.09	0.962 ± 0.011	0.955 ± 0.011	0.86 ± 0.05	0.86 ± 0.02
**300**	5.3 ± 0.3	5.24 ± 0.14	0.84 ± 0.02	0.831 ± 0.011	0.72 ± 0.03	0.708 ± 0.014
**600**	3.0 ± 0.2	3.12 ± 0.16	0.62 ± 0.03	0.63 ± 0.02	0.44 ± 0.03	0.46 ± 0.02
**1000**	1.88 ± 0.11	2.06 ± 0.12	0.44 ± 0.02	0.47 ± 0.02	0.269 ± 0.017	0.297 ± 0.020
**5000**	6.9 ± 0.3	7.0 ± 0.2	0.919 ± 0.013	0.923 ± 0.008	0.83 ± 0.02	0.839 ± 0.013
**10000**	3.93 ± 0.14	4.11 ± 0.16	0.72 ± 0.02	0.739 ± 0.019	0.55 ± 0.03	0.57 ± 0.02

Supplementary Table S10. Mean value of the ICSDs with modified options in Geant4-DNA using the common interaction cross sections data set and the same differential elastic cross sections (those originally implemented in option 2, Champion's model). Therefore, the only difference between all those "options" are the differential ionization cross sections and angular distributions in the ionization process.

**Energy (eV)**	G4DNA-**2** with common cross sections	G4DNA-**4** with common cross sections	G4DNA-**4** with common cross sections and differential cross-sections of elastic processes from option 2	G4DNA-**6** with common cross sections	G4DNA-**6** with common cross sections and differential cross-sections of elastic process from option 2
**20**	0.5975 ± 0.0016	0.6123 ± 0.0015	0.6110 ± 0.0015	0.6088 ± 0.0015	0.6135 ± 0.0015
**50**	1.824 ± 0.002	1.836 ± 0.002	1.858 ± 0.002	1.936 ± 0.002	1.950 ± 0.002
**100**	3.751 ± 0.003	3.701 ± 0.004	3.798 ± 0.003	3.882 ± 0.004	3.964 ± 0.004
**300**	5.710 ± 0.012	5.258 ± 0.012	5.808 ± 0.012	5.235 ± 0.013	5.255 ± 0.012
**600**	3.374 ± 0.010	3.234 ± 0.009	3.391 ± 0.010	2.841 ± 0.008	2.965 ± 0.009
**1000**	2.308 ± 0.008	2.290 ± 0.007	2.317 ± 0.007	2.040 ± 0.007	2.066 ± 0.007
**5000**	7.208 ± 0.016	7.285 ± 0.016	6.930 ± 0.017	6.364 ± 0.014	6.185 ± 0.015
**10000**	4.344 ± 0.013	4.352 ± 0.013	4.242 ± 0.013	3.908 ± 0.012	3.868 ± 0.012

Participants' data with modified codes

Supplementary Table S11. Participants data on ICSDs with modified codes (limited to 28 ionizations).

Energy (eV)	ICS	G4DNA- 2	G4DNA- 4	G4DNA- 6	PARTRAC	MCwater	PTra	PHITS
20	0	4.03E-01	3.88E-01	3.91E-01	3.79E-01	3.97E-01	3.92E-01	3.78E-01
20	1	5.98E-01	6.12E-01	6.09E-01	6.21E-01	6.03E-01	6.09E-01	6.22E-01
50	0	1.67E-02	2.21E-02	2.18E-02	1.78E-02	9.10E-02	2.59E-02	1.63E-02
50	1	2.81E-01	2.80E-01	2.38E-01	2.64E-01	2.94E-01	2.93E-01	2.18E-01
50	2	5.64E-01	5.41E-01	5.25E-01	5.63E-01	4.45E-01	5.76E-01	5.46E-01
50	3	1.38E-01	1.55E-01	2.13E-01	1.55E-01	1.68E-01	1.05E-01	2.17E-01
50	4	7.00E-04	2.90E-03	2.70E-03	7.00E-04	2.10E-03	0.00E+00	3.07E-03
100	0	6.30E-03	8.00E-03	8.40E-03	6.20E-03	2.65E-02	1.41E-02	6.36E-03
100	1	2.34E-02	3.06E-02	2.86E-02	1.78E-02	7.50E-02	4.11E-02	2.07E-02
100	2	8.02E-02	9.97E-02	8.38E-02	7.30E-02	1.54E-01	1.14E-01	6.27E-02
100	3	2.66E-01	2.61E-01	2.20E-01	2.53E-01	2.48E-01	2.96E-01	2.02E-01
100	4	3.86E-01	3.60E-01	3.43E-01	3.88E-01	2.78E-01	3.70E-01	3.65E-01
100	5	2.05E-01	1.98E-01	2.49E-01	2.22E-01	1.76E-01	1.53E-01	2.73E-01
100	6	3.24E-02	4.09E-02	6.24E-02	3.87E-02	4.09E-02	1.22E-02	6.66E-02
100	7	1.10E-03	1.80E-03	4.70E-03	1.30E-03	1.81E-03	4.00E-05	3.42E-03
100	8	0.00E+00	0.00E+00	0.00E+00	0.00E+00	0.00E+00	0.00E+00	1.47E-05
300	0	4.35E-02	4.52E-02	6.60E-02	7.83E-02	7.23E-02	7.85E-02	5.19E-02
300	1	8.77E-02	1.02E-01	1.20E-01	1.07E-01	9.27E-02	1.30E-01	1.07E-01
300	2	1.13E-01	1.24E-01	1.29E-01	1.19E-01	1.03E-01	1.39E-01	1.32E-01
300	3	1.15E-01	1.22E-01	1.24E-01	1.13E-01	1.04E-01	1.26E-01	1.30E-01
300	4	1.03E-01	1.09E-01	1.01E-01	9.73E-02	9.47E-02	1.06E-01	1.11E-01
300	5	8.01E-02	9.61E-02	8.51E-02	8.29E-02	8.63E-02	8.53E-02	9.11E-02
300	6	7.96E-02	7.65E-02	6.97E-02	6.97E-02	7.70E-02	6.79E-02	7.34E-02
300	7	6.52E-02	6.68E-02	5.14E-02	5.68E-02	7.03E-02	5.55E-02	5.79E-02
300	8	5.36E-02	4.94E-02	4.46E-02	5.01E-02	6.31E-02	4.65E-02	4.75E-02
300	9	5.38E-02	4.81E-02	3.46E-02	4.50E-02	5.90E-02	4.20E-02	3.94E-02
300	10	5.20E-02	3.97E-02	3.30E-02	4.34E-02	5.31E-02	4.01E-02	3.51E-02
300	11	5.34E-02	4.50E-02	2.64E-02	4.30E-02	4.49E-02	3.50E-02	3.33E-02
300	12	4.90E-02	3.51E-02	2.71E-02	4.02E-02	3.50E-02	2.64E-02	3.11E-02
300	13	2.81E-02	2.38E-02	2.74E-02	3.02E-02	2.31E-02	1.46E-02	2.68E-02
300	14	1.46E-02	1.04E-02	2.39E-02	1.65E-02	1.34E-02	5.62E-03	1.86E-02
300	15	6.70E-03	5.10E-03	1.76E-02	6.80E-03	5.70E-03	1.36E-03	9.32E-03
300	16	1.40E-03	1.30E-03	1.18E-02	1.90E-03	1.73E-03	2.10E-04	3.34E-03
300	17	1.00E-04	2.00E-04	5.50E-03	3.00E-04	4.60E-04	5.00E-05	7.78E-04
300	18	1.00E-04	1.00E-04	1.30E-03	0.00E+00	5.00E-05	0.00E+00	8.94E-05
300	19	0.00E+00	0.00E+00	1.00E-04	0.00E+00	1.00E-05	0.00E+00	8.65E-06
600	0	1.08E-01	1.14E-01	1.12E-01	2.20E-01	1.87E-01	2.18E-01	1.43E-01
600	1	2.14E-01	2.08E-01	2.43E-01	1.95E-01	1.68E-01	2.33E-01	1.95E-01
600	2	1.77E-01	1.83E-01	2.11E-01	1.66E-01	1.52E-01	1.77E-01	1.70E-01
600	3	1.44E-01	1.46E-01	1.47E-01	1.23E-01	1.26E-01	1.24E-01	1.29E-01
600	4	9.87E-02	1.04E-01	1.00E-01	8.55E-02	9.64E-02	7.94E-02	9.09E-02
600	5	7.39E-02	7.32E-02	6.12E-02	5.93E-02	7.04E-02	5.32E-02	6.37E-02

Energy (eV)	ICS	G4DNA- 2	G4DNA- 4	G4DNA- 6	PARTRAC	MCwater	PTra	PHITS
600	6	5.20E-02	5.07E-02	4.21E-02	4.26E-02	5.19E-02	3.53E-02	4.62E-02
600	7	3.80E-02	3.78E-02	2.75E-02	3.00E-02	3.90E-02	2.45E-02	3.47E-02
600	8	2.35E-02	2.51E-02	1.59E-02	2.18E-02	2.84E-02	1.56E-02	2.60E-02
600	9	1.99E-02	1.60E-02	1.25E-02	1.47E-02	2.06E-02	1.16E-02	2.03E-02
600	10	1.33E-02	1.04E-02	6.90E-03	1.07E-02	1.50E-02	8.02E-03	1.53E-02
600	11	9.90E-03	9.20E-03	7.50E-03	8.10E-03	1.08E-02	5.80E-03	1.31E-02
600	12	7.20E-03	6.10E-03	3.80E-03	5.90E-03	8.39E-03	3.77E-03	1.00E-02
600	13	5.10E-03	5.20E-03	2.00E-03	4.10E-03	6.97E-03	2.86E-03	8.27E-03
600	14	4.10E-03	3.50E-03	2.40E-03	3.40E-03	4.67E-03	2.10E-03	6.97E-03
600	15	2.60E-03	1.80E-03	1.10E-03	2.50E-03	3.95E-03	1.57E-03	5.49E-03
600	16	2.60E-03	1.60E-03	1.10E-03	1.80E-03	2.78E-03	9.40E-04	4.50E-03
600	17	1.50E-03	1.20E-03	1.00E-03	1.50E-03	2.23E-03	8.10E-04	3.70E-03
600	18	2.10E-03	9.00E-04	7.00E-04	1.10E-03	1.43E-03	5.30E-04	3.09E-03
600	19	7.00E-04	4.00E-04	2.00E-04	7.00E-04	1.41E-03	3.00E-04	2.35E-03
600	20	7.00E-04	7.00E-04	3.00E-04	7.00E-04	1.01E-03	4.60E-04	2.06E-03
600	21	6.00E-04	5.00E-04	1.00E-04	5.00E-04	6.90E-04	2.50E-04	1.55E-03
600	22	3.00E-04	0.00E+00	3.00E-04	3.00E-04	7.30E-04	2.20E-04	1.33E-03
600	23	5.00E-04	2.00E-04	0.00E+00	3.00E-04	3.10E-04	5.00E-05	1.01E-03
600	24	3.00E-04	1.00E-04	1.00E-04	3.00E-04	2.20E-04	6.00E-05	7.93E-04
600	25	2.00E-04	1.00E-04	0.00E+00	2.00E-04	2.20E-04	7.00E-05	6.47E-04
600	26	0.00E+00	0.00E+00	1.00E-04	2.00E-04	9.00E-05	1.00E-05	4.15E-04
600	27	0.00E+00	0.00E+00	0.00E+00	1.00E-04	5.00E-05	0.00E+00	3.02E-04
600	28	0.00E+00	0.00E+00	0.00E+00	0.00E+00	0.00E+00	1.00E-05	1.51E-04
1 000	0	1.77E-01	1.78E-01	1.82E-01	3.62E-01	2.89E-01	3.61E-01	2.74E-01
1 000	1	2.99E-01	2.96E-01	3.22E-01	2.22E-01	2.06E-01	2.65E-01	2.60E-01
1 000	2	1.91E-01	1.83E-01	2.14E-01	1.56E-01	1.58E-01	1.56E-01	1.73E-01
1 000	3	1.21E-01	1.30E-01	1.16E-01	9.45E-02	1.13E-01	8.84E-02	1.05E-01
1 000	4	7.76E-02	7.95E-02	6.79E-02	5.89E-02	7.71E-02	5.06E-02	6.25E-02
1 000	5	4.52E-02	4.88E-02	3.76E-02	3.63E-02	5.19E-02	2.87E-02	3.98E-02
1 000	6	3.09E-02	3.01E-02	2.30E-02	2.36E-02	3.49E-02	1.72E-02	2.49E-02
1 000	7	1.82E-02	2.02E-02	1.47E-02	1.51E-02	2.29E-02	1.23E-02	1.73E-02
1 000	8	1.38E-02	1.11E-02	7.40E-03	1.03E-02	1.52E-02	7.30E-03	1.16E-02
1 000	9	8.40E-03	6.60E-03	5.70E-03	6.80E-03	1.03E-02	4.47E-03	8.86E-03
1 000	10	5.90E-03	5.30E-03	2.80E-03	4.40E-03	7.11E-03	2.89E-03	5.94E-03
1 000	11	3.90E-03	3.90E-03	2.90E-03	2.90E-03	4.75E-03	1.68E-03	4.06E-03
1 000	12	1.90E-03	2.20E-03	1.50E-03	2.00E-03	3.36E-03	1.23E-03	2.95E-03
1 000	13	1.80E-03	1.10E-03	6.00E-04	1.60E-03	2.46E-03	8.90E-04	2.49E-03
1 000	14	1.50E-03	1.30E-03	1.00E-03	1.00E-03	1.46E-03	5.30E-04	1.78E-03
1 000	15	1.00E-03	7.00E-04	5.00E-04	7.00E-04	1.17E-03	3.30E-04	1.22E-03
1 000	16	4.00E-04	7.00E-04	5.00E-04	4.00E-04	5.90E-04	2.60E-04	9.53E-04
1 000	17	5.00E-04	4.00E-04	1.00E-04	3.00E-04	4.90E-04	1.50E-04	7.37E-04
1 000	18	4.00E-04	4.00E-04	1.00E-04	4.00E-04	4.10E-04	1.40E-04	6.29E-04
1 000	19	2.00E-04	3.00E-04	2.00E-04	1.00E-04	1.60E-04	3.00E-05	3.42E-04
1 000	20	1.00E-04	1.00E-04	1.00E-04	1.00E-04	2.10E-04	3.00E-05	3.87E-04

Energy (eV)	ICS	G4DNA- 2	G4DNA- 4	G4DNA- 6	PARTRAC	MCwater	PTra	PHITS
1 000	21	1.00E-04	1.00E-04	2.00E-04	0.00E+00	1.00E-04	3.00E-05	4.05E-04
1 000	22	2.00E-04	0.00E+00	2.00E-04	0.00E+00	8.00E-05	1.00E-05	3.06E-04
1 000	23	2.00E-04	0.00E+00	0.00E+00	0.00E+00	1.00E-04	2.00E-05	1.62E-04
1 000	24	1.00E-04	1.00E-04	0.00E+00	0.00E+00	6.00E-05	2.00E-05	1.44E-04
1 000	25	2.00E-04	0.00E+00	0.00E+00	0.00E+00	5.00E-05	2.00E-05	1.26E-04
1 000	26	0.00E+00	1.00E-04	0.00E+00	0.00E+00	0.00E+00	0.00E+00	1.08E-04
1 000	27	0.00E+00	0.00E+00	0.00E+00	0.00E+00	0.00E+00	1.00E-05	2.70E-05
1 000	28	0.00E+00	0.00E+00	0.00E+00	0.00E+00	0.00E+00	0.00E+00	3.60E-05
5 000	0	1.60E-02	1.57E-02	1.63E-02	3.11E-02	2.53E-02	3.18E-02	1.44E-02
5 000	1	5.11E-02	4.98E-02	5.59E-02	6.30E-02	5.01E-02	7.80E-02	3.97E-02
5 000	2	7.44E-02	7.58E-02	9.62E-02	8.52E-02	7.27E-02	1.09E-01	7.43E-02
5 000	3	1.01E-01	1.00E-01	1.20E-01	9.63E-02	8.53E-02	1.22E-01	9.40E-02
5 000	4	1.03E-01	9.87E-02	1.17E-01	9.61E-02	8.88E-02	1.17E-01	9.96E-02
5 000	5	9.11E-02	9.56E-02	1.14E-01	9.11E-02	8.94E-02	1.02E-01	9.75E-02
5 000	6	8.96E-02	8.92E-02	9.32E-02	8.04E-02	8.29E-02	8.47E-02	9.05E-02
5 000	7	7.95E-02	7.51E-02	7.49E-02	6.75E-02	7.49E-02	6.72E-02	7.92E-02
5 000	8	6.29E-02	6.52E-02	5.49E-02	5.75E-02	6.46E-02	5.25E-02	6.73E-02
5 000	9	5.36E-02	5.76E-02	4.73E-02	4.76E-02	5.51E-02	4.11E-02	5.27E-02
5 000	10	4.32E-02	3.79E-02	3.44E-02	3.88E-02	4.64E-02	3.26E-02	4.39E-02
5 000	11	3.44E-02	3.50E-02	2.67E-02	3.14E-02	3.70E-02	2.52E-02	3.43E-02
5 000	12	2.51E-02	2.86E-02	2.02E-02	2.58E-02	3.08E-02	2.07E-02	2.59E-02
5 000	13	2.15E-02	2.04E-02	1.79E-02	2.20E-02	2.57E-02	1.73E-02	2.32E-02
5 000	14	2.01E-02	1.73E-02	1.43E-02	1.73E-02	2.09E-02	1.33E-02	1.84E-02
5 000	15	1.59E-02	1.54E-02	1.07E-02	1.48E-02	1.76E-02	1.09E-02	1.51E-02
5 000	16	1.30E-02	1.42E-02	1.07E-02	1.22E-02	1.49E-02	9.72E-03	1.32E-02
5 000	17	9.40E-03	1.31E-02	6.90E-03	1.05E-02	1.27E-02	7.91E-03	9.76E-03
5 000	18	9.50E-03	8.30E-03	6.20E-03	9.80E-03	1.05E-02	6.29E-03	9.32E-03
5 000	19	7.50E-03	7.70E-03	5.20E-03	7.80E-03	9.31E-03	5.32E-03	6.94E-03
5 000	20	5.30E-03	7.00E-03	4.10E-03	6.90E-03	8.51E-03	4.75E-03	6.63E-03
5 000	21	5.30E-03	5.60E-03	3.80E-03	6.60E-03	6.80E-03	3.93E-03	5.15E-03
5 000	22	5.30E-03	5.00E-03	3.10E-03	5.60E-03	6.11E-03	3.57E-03	4.97E-03
5 000	23	3.90E-03	4.80E-03	3.30E-03	5.40E-03	5.11E-03	3.05E-03	3.85E-03
5 000	24	3.20E-03	4.20E-03	3.10E-03	5.40E-03	4.93E-03	2.85E-03	3.94E-03
5 000	25	4.00E-03	3.00E-03	2.60E-03	4.30E-03	4.46E-03	2.30E-03	3.27E-03
5 000	26	3.50E-03	2.90E-03	2.10E-03	4.20E-03	3.74E-03	2.21E-03	3.99E-03
5 000	27	3.60E-03	3.50E-03	1.70E-03	3.80E-03	3.65E-03	2.00E-03	2.78E-03
5 000	28	2.90E-03	3.80E-03	1.70E-03	3.20E-03	3.25E-03	1.98E-03	2.73E-03
10 000	0	6.58E-02	6.48E-02	6.60E-02	1.48E-01	1.09E-01	1.49E-01	8.95E-02
10 000	1	1.64E-01	1.59E-01	1.73E-01	1.59E-01	1.34E-01	1.95E-01	1.54E-01
10 000	2	1.63E-01	1.68E-01	1.93E-01	1.53E-01	1.39E-01	1.76E-01	1.70E-01
10 000	3	1.45E-01	1.39E-01	1.56E-01	1.25E-01	1.23E-01	1.34E-01	1.44E-01
10 000	4	1.11E-01	1.14E-01	1.15E-01	9.64E-02	1.05E-01	9.60E-02	1.09E-01
10 000	5	8.54E-02	8.51E-02	7.75E-02	6.92E-02	8.44E-02	6.57E-02	8.00E-02
10 000	6	6.09E-02	6.27E-02	5.40E-02	5.12E-02	6.42E-02	4.42E-02	5.49E-02

Energy (eV)	ICS	G4DNA- 2	G4DNA- 4	G4DNA- 6	PARTRAC	MCwater	PTra	PHITS
10 000	7	3.75E-02	4.29E-02	3.89E-02	3.74E-02	4.88E-02	3.13E-02	4.04E-02
10 000	8	3.32E-02	3.07E-02	2.62E-02	2.72E-02	3.74E-02	2.22E-02	2.94E-02
10 000	9	2.04E-02	2.35E-02	1.68E-02	2.04E-02	2.79E-02	1.58E-02	2.16E-02
10 000	10	2.03E-02	1.87E-02	1.09E-02	1.55E-02	2.03E-02	1.15E-02	1.64E-02
10 000	11	1.24E-02	1.33E-02	9.30E-03	1.21E-02	1.61E-02	9.72E-03	1.24E-02
10 000	12	1.16E-02	1.03E-02	7.60E-03	1.05E-02	1.27E-02	7.12E-03	9.24E-03
10 000	13	9.70E-03	7.60E-03	7.50E-03	8.10E-03	9.91E-03	6.07E-03	7.74E-03
10 000	14	6.20E-03	5.40E-03	5.00E-03	6.30E-03	7.87E-03	4.45E-03	6.48E-03
10 000	15	5.70E-03	5.10E-03	4.80E-03	5.70E-03	6.72E-03	3.82E-03	5.34E-03
10 000	16	4.20E-03	4.20E-03	3.80E-03	4.80E-03	5.51E-03	2.87E-03	3.98E-03
10 000	17	3.00E-03	3.70E-03	3.20E-03	3.70E-03	4.86E-03	3.01E-03	3.33E-03
10 000	18	2.50E-03	4.20E-03	1.30E-03	3.50E-03	3.78E-03	2.15E-03	2.72E-03
10 000	19	3.70E-03	2.80E-03	1.80E-03	3.00E-03	3.43E-03	1.97E-03	2.76E-03
10 000	20	2.50E-03	3.10E-03	1.40E-03	2.70E-03	2.86E-03	1.68E-03	2.22E-03
10 000	21	2.80E-03	1.50E-03	1.80E-03	2.70E-03	2.62E-03	1.48E-03	1.68E-03
10 000	22	1.50E-03	1.30E-03	1.80E-03	2.40E-03	2.36E-03	1.27E-03	1.68E-03
10 000	23	1.20E-03	1.40E-03	1.10E-03	2.10E-03	2.30E-03	1.16E-03	1.65E-03
10 000	24	1.30E-03	1.30E-03	1.50E-03	2.20E-03	1.95E-03	9.80E-04	1.76E-03
10 000	25	1.90E-03	1.50E-03	1.10E-03	2.30E-03	1.86E-03	9.00E-04	1.04E-03
10 000	26	1.80E-03	2.90E-03	1.20E-03	2.10E-03	1.68E-03	8.70E-04	1.47E-03
10 000	27	1.50E-03	1.50E-03	1.40E-03	1.80E-03	1.47E-03	8.80E-04	1.40E-03
10 000	28	1.50E-03	1.20E-03	1.20E-03	1.50E-03	1.36E-03	6.90E-04	1.54E-03

Supplementary Figures

Original codes cross sections and common-set cross section data

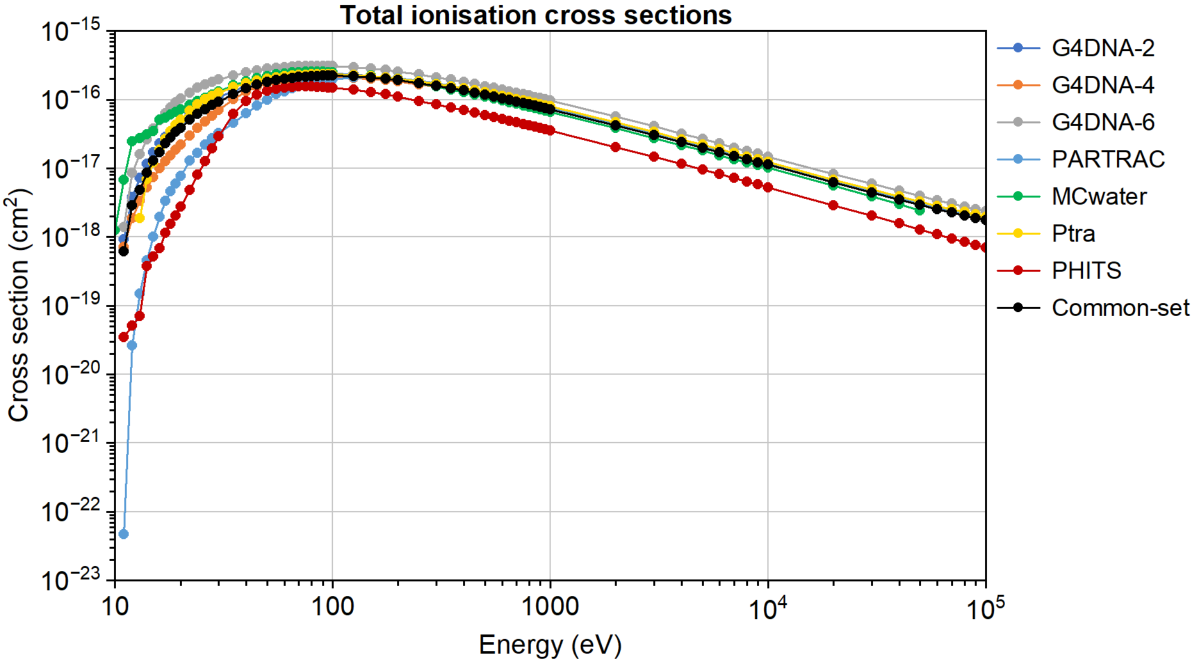

Supplementary Figure S1. Total ionization cross sections originally used in the contributing MCTS codes. The data in pink is the total ionization cross section used in the common-set cross sections for modifying the codes. The common-set cross sections were calculated as indicated in the materials and methods section.

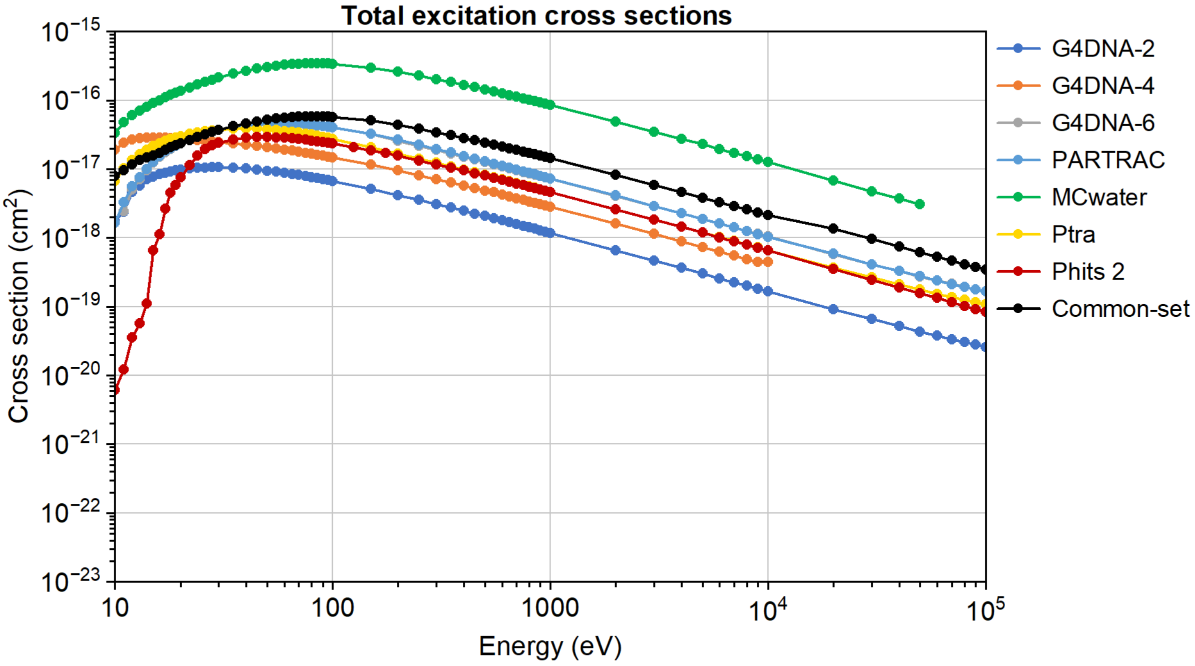

Supplementary Figure S2. Total excitation cross sections of the MCTS codes used by the contributors. In pink, the total excitation cross section is shown that was used in the common-set cross sections for modifying the codes. The common set cross sections were calculated as indicated in the materials and methods section.

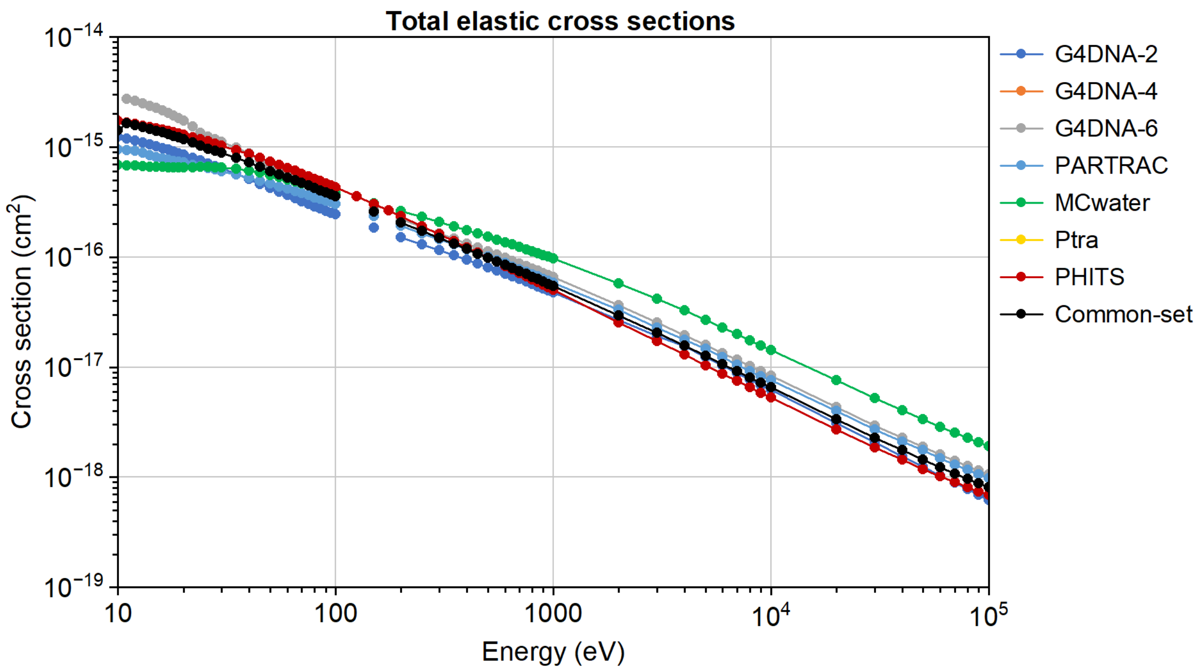

Supplementary Figure S3. Total elastic cross sections of the MCTS codes used by the contributors. In pink, the total elastic cross section is shown that was used in the common-set cross sections for modifying the codes. The common set cross sections were calculated as indicated in the materials and methods section.

Illustration of basic elements in MCTS codes

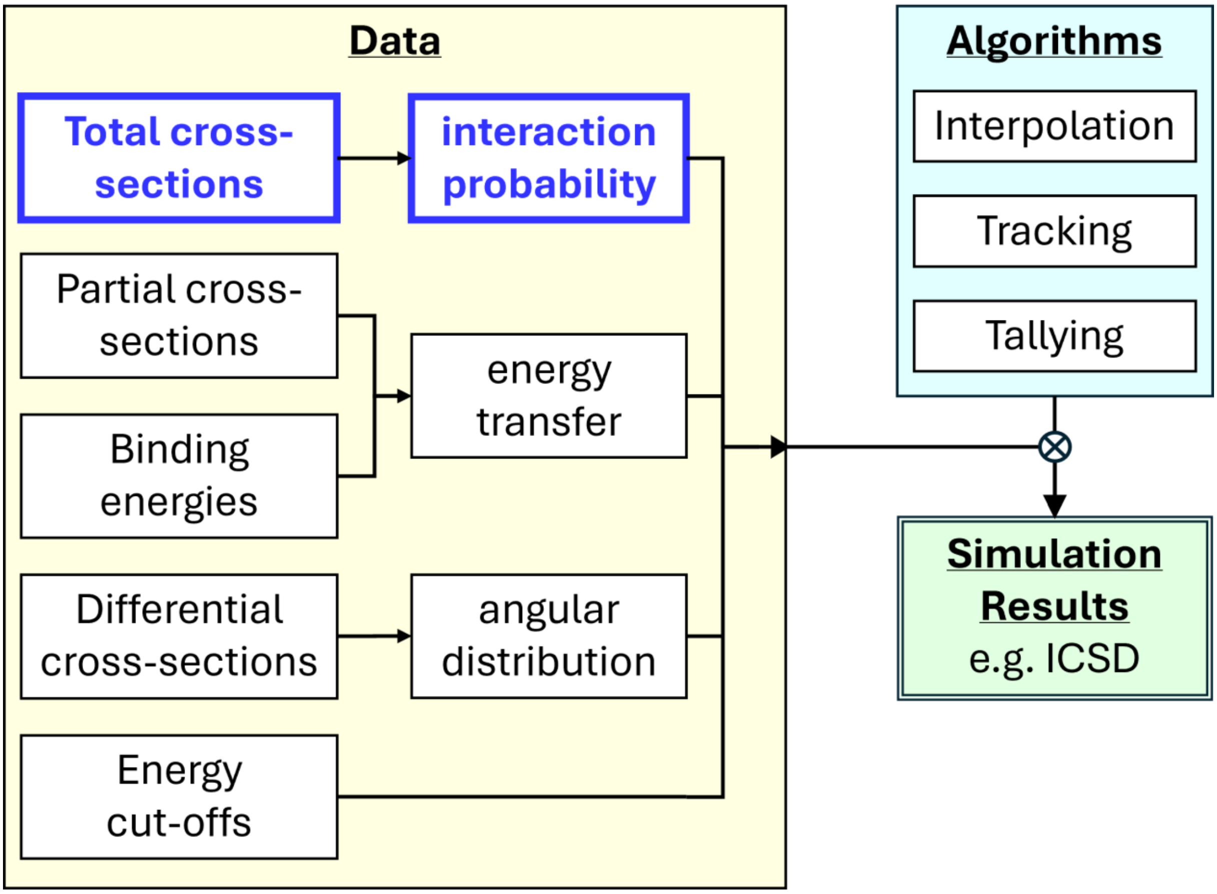

Supplementary Figure S4. Schematic view of the different elements comprising the MCTS codes that come into play when calculating ICSDs. The total cross-sections whose contribution to the variability of results was studied in this work are identified in blue

ICSDs of original MCTS codes versus those obtained with modified codes

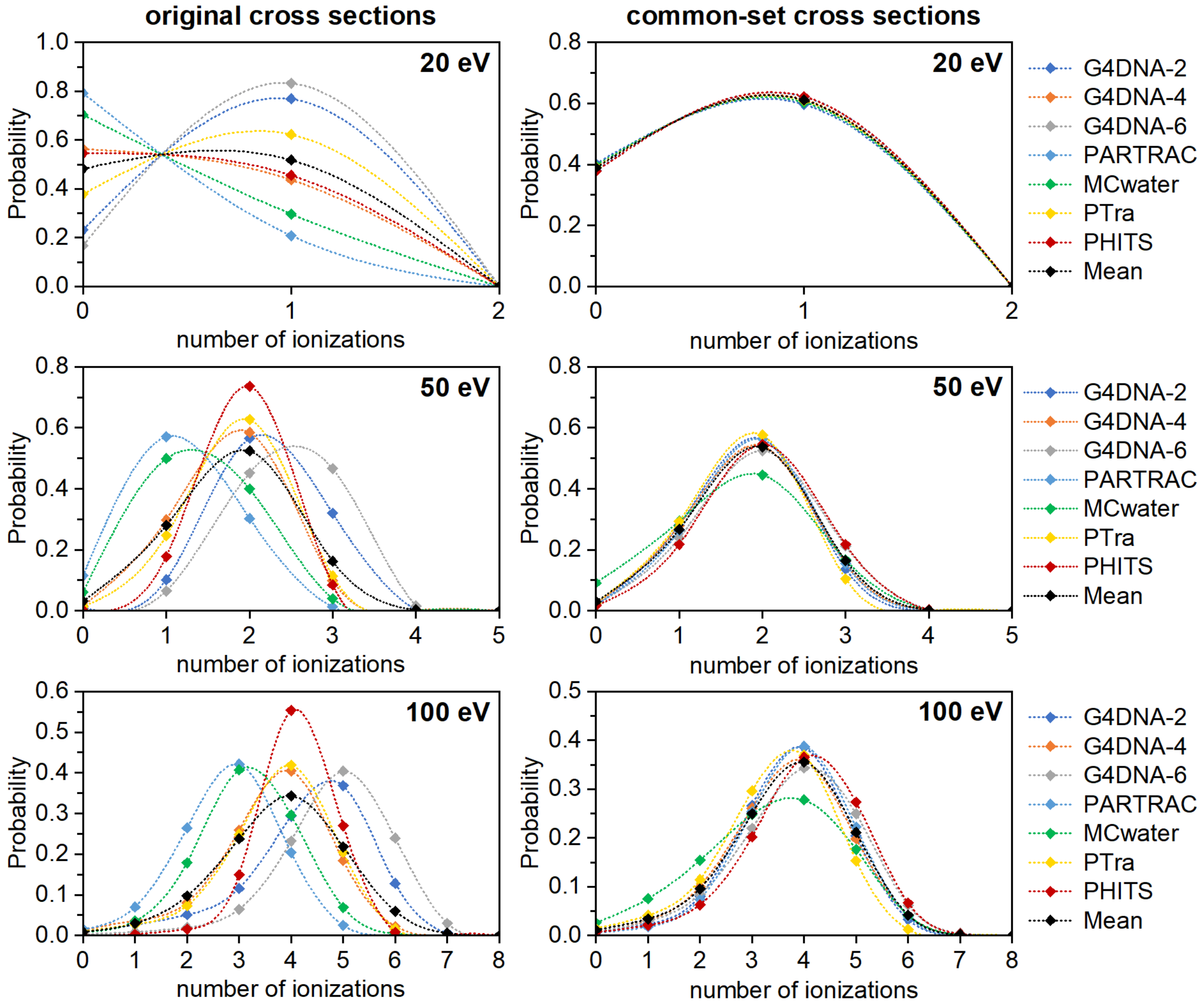

Supplementary Figure S5. ICSDs obtained with original MCTS codes compared to those obtained with modified codes using the cross section common data set for electrons of 20 eV, 50 eV and 100 eV. Note: the lines are only there to guide the eye, as non-integer values for the number of ionizations are meaningless.

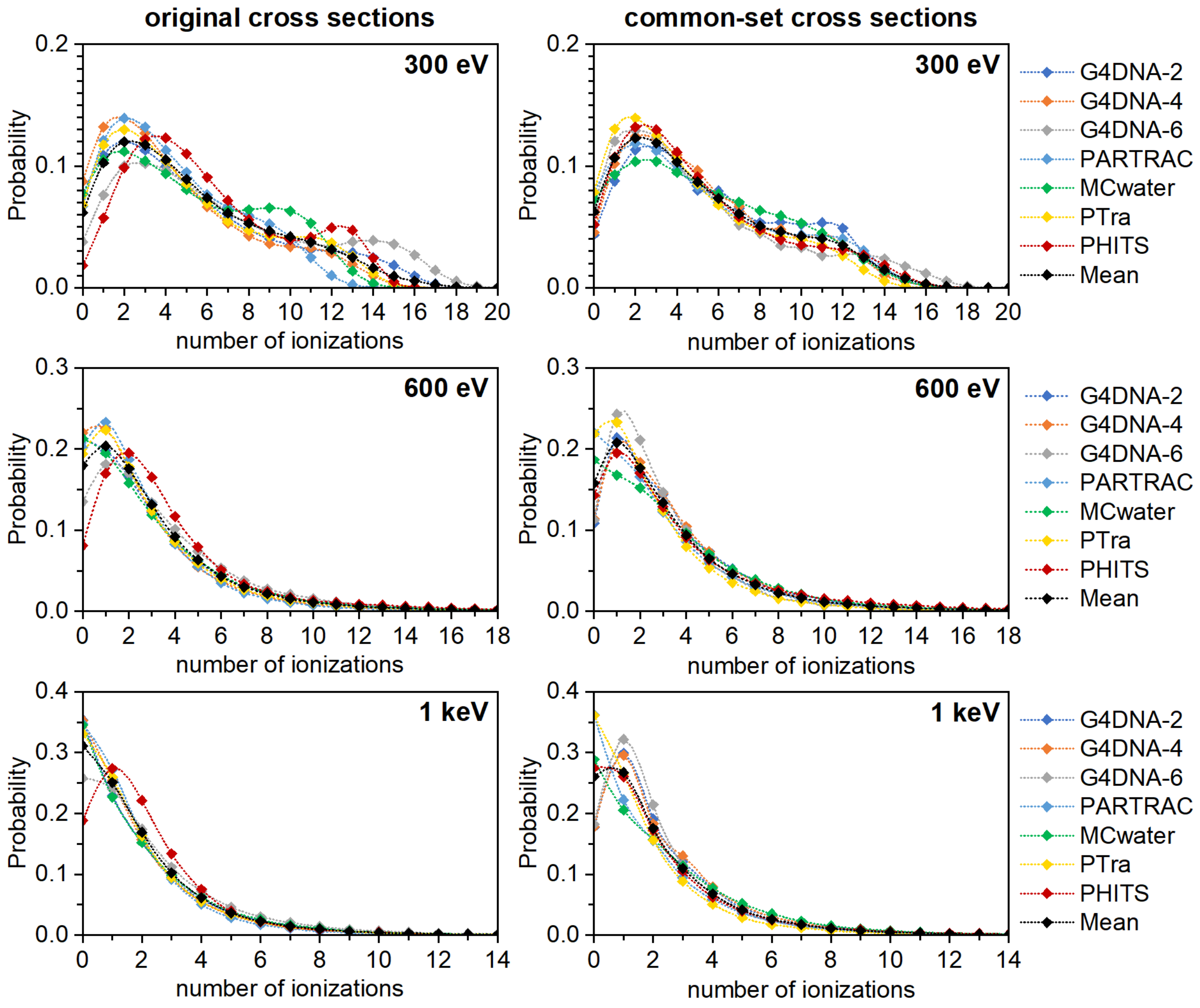

Supplementary Figure S6. ICSDs obtained with original MCTS codes compared to those obtained with modified codes using the cross section common data set for electrons of 300 eV, 600 eV and 1 keV. Note: the lines are only there to guide the eye, as non-integer values for the number of ionizations are meaningless.

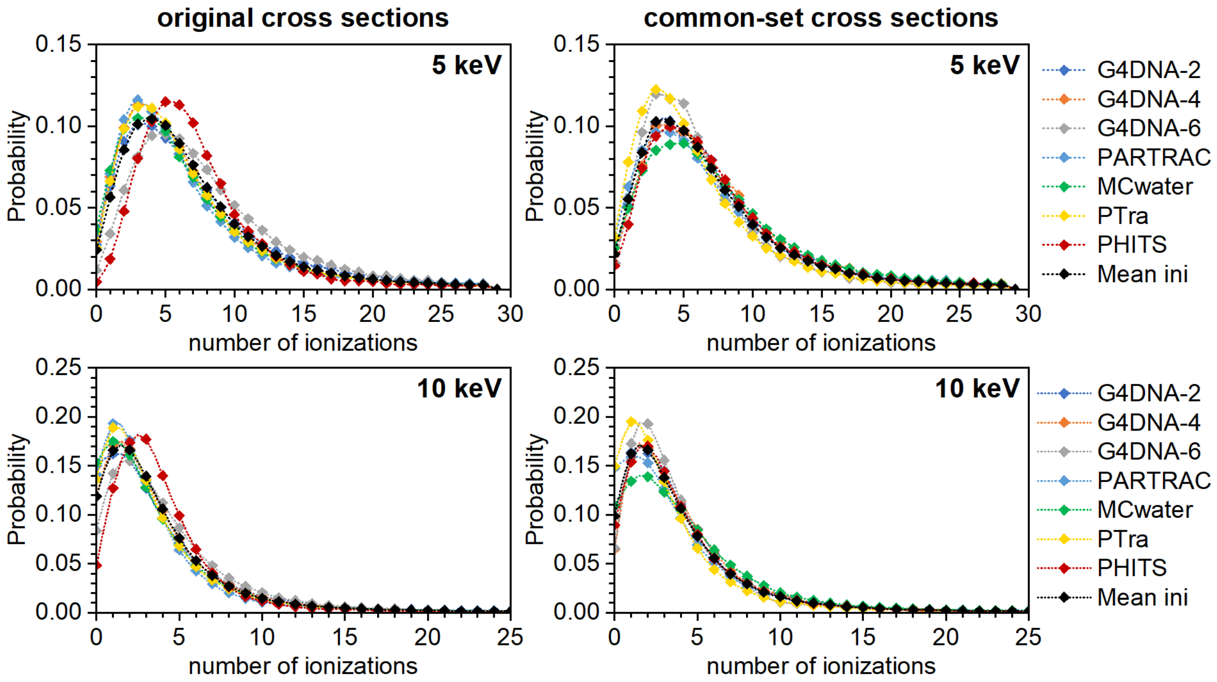

Supplementary Figure S7. ICSDs obtained with original MCTS codes compared to those obtained with modified codes using the cross section common data set for electrons of 5 keV and 10 keV. Note: the lines are only there to guide the eye, as non-integer values for the number of ionizations are meaningless.

Modification of Geant4-DNA options: common cross section data set and equal differential elastic cross sections

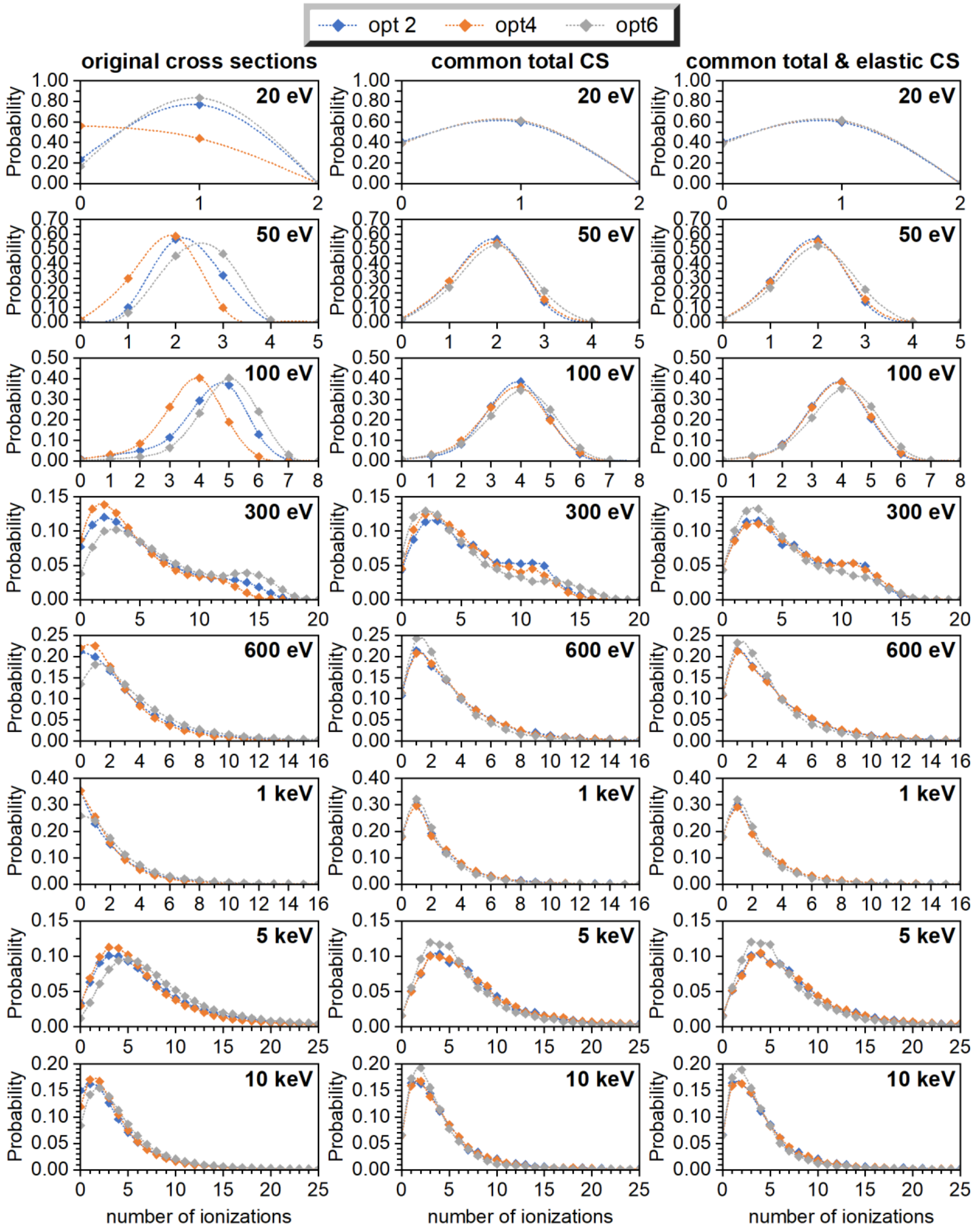

Supplementary Figure S8. ICSDs obtained using Geant4-DNA options 2, 4 and 6 with original cross sections (left) compared to the ones obtained with the common cross section data set (center) and same differential elastic cross sections (those implemented in option 2 in the original code, Model from Champion) and different differential ionization cross sections (originally in options 2, 4 and 6) (right). Note: the lines are only there to guide the eye, as non-integer values for the number of ionizations are meaningless.

Supplementary Information: code availability

Some of the codes used in this study are openly available as general-purpose toolkits (Geant4-DNA versions, PHITS) with documentation and tutorials posted on the respective websites of the developer communities and regularly offered training courses for different proficiency levels. The other codes are developed within a small group of experts, and their proficient use requires advanced expertise. Therefore, these codes can only be made available to interested parties in the frame of collaborations with the developers with intense training.

Snapshots of the codes, input files, and user applications used in this study have been archived in repositories, along with procedures for their use, for example in independent replication of the study. They are available under request:

- Geant4-DNA options 2, 4 and 6 are publicly available by downloading the Geant4 code (https://geant4.org). In this work, version 11.2.0 was used, but other versions containing these options can be used. The application developed for this work and the corresponding instructions are available here: https://gitlab.asnr.fr/plosone_icsd/icsd. Contact: Yann Perrot <yann.perrot@asnr.fr> and Carmen Villagrasa <carmen.villagrasa@asnr.f>
- MCWater: both original and modified applications and input files are stored in https://drive.google.com/file/d/1fliY9qs1ZGC4_kbds4mZNfm8lRdw2lDm/view?usp=sharing. Contact: Zine-El-Abidine Chaoui <zchaoui@univ-setif.dz>.
- PARTRAC: https://github.com/kundrat-ujf/PARTRAC-EURADOS-WG6-ICDS-intercomparison. Contact: Pavel Kundrát <kundrat@ujf.cas.cz> and Giorgio Baiocco <giorgio.baiocco@unipv.it>.
- PHITS: For this exercise, code versions were derived from PHITS version 3.34, which differ from the normal operation of PHITS in that autoionization was suppressed. To get access to the codes, users must first register as PHITS users. Please see the PHITS website for details (https://phits.jaea.go.jp/howtoget.html) and contact the authors for the two dedicated code versions (Takeshi Kai <kai.takeshi@jaea.go.jp> and Yusuke Matsuya <ymatsuya.radiation@gmail.com>) to verify proficiency for use of the code. Then, to repeat the simulations presented in this work download the PHITS package after requesting it from the PHITS office (https://phits.jaea.go.jp/contact/edit/en) and follow the instruction in it for the original and modified version.
- PTra: https://gitlab.com/track-nanodosimetry/ptra-h2o. Contact: Marcin Pietrzak <marcin.pietrzak@ifj.edu.pl> and Hans Rabus <hans.rabus@ptb.de>.

In any case, if future use is made of the codes, this may only be declared as a replication study, if the codes are used as provided. If changes to the codes (or the relevant input cards, parameter settings etc.) are made, this must be clearly indicated in any report on the results obtained.
